# Separation of Metabolites and Macromolecules for Short-TE ^1^H-MRSI Using Learned Component-Specific Representations

**DOI:** 10.1109/TMI.2020.3048933

**Published:** 2021-04-01

**Authors:** Yahang Li, Zepeng Wang, Ruoyu Sun, Fan Lam

**Affiliations:** Department of Bioengineering, University of Illinois Urbana–Champaign, Urbana, IL 61801 USA.; Department of Industrial and Enterprise Systems Engineering, University of Illinois Urbana–Champaign, Urbana, IL 61801 USA.; Department of Bioengineering, University of Illinois Urbana–Champaign, Urbana, IL 61801 USA; Beckman Institute for Advanced Science and Technology, University of Illinois Urbana–Champaign, Urbana, IL 61801 USA

**Keywords:** Proton (^1^H) magnetic resonance spectroscopic imaging, short TE, signal separation, deep learning, deep autoencoder, low-dimensional models

## Abstract

Short-echo-time (TE) proton magnetic resonance spectroscopic imaging (MRSI) allows for simultaneously mapping a number of molecules in the brain, and has been recognized as an important tool for studying in vivo biochemistry in various neuroscience and disease applications. However, separation of the metabolite and macromolecule (MM) signals present in the short-TE data with significant spectral overlaps remains a major technical challenge. This work introduces a new approach to solve this problem by integrating imaging physics and representation learning. Specifically, a mixed unsupervised and supervised learning-based strategy was developed to learn the metabolite and MM-specific low-dimensional representations using deep autoencoders. A constrained reconstruction formulation is proposed to integrate the MRSI spatiospectral encoding model and the learned representations as effective constraints for signal separation. An efficient algorithm was developed to solve the resulting optimization problem with provable convergence. Simulation and experimental results have been obtained to demonstrate the component-specific representation power of the learned models and the capability of the proposed method in separating metabolite and MM signals for practical short-TE ^1^H-MRSI data.

## I. Introduction

PROTON MRSI (^1^H-MRSI) is a unique molecular imaging modality that can noninvasively map various endogenous metabolites in the brain. This molecular-level information has been demonstrated useful in different neuroscience and clinical applications, including brain tumors [1], [2], metabolic disorders [3], and neurodegenerative diseases [4], [5]. Short-echo-time (TE) ^1^H-MRSI, in particular, offers several unique advantages compared to the more commonly used long-TE acquisitions, such as higher signal-to-noise ratio (SNR) due to less relaxation-induced signal loss and improved detection and quantification of molecules with short *T*_2_’s and/or *J*-coupled spins, e.g., myo-inositol (mI), glutamate (Glu) and glutamine (Gln) [6]–[10]. However, the applications of short-TE ^1^H-MRSI have been limited by several technical challenges. One of these major challenges is the presence of macromolecule (MM) signals that overlap with the metabolite signals across the entire spectrum. This makes accurate and reproducible metabolite quantification difficult. It has been demonstrated that metabolite quantification can be substantially improved with better characterization and separation of MM signals from the short-TE data [11]–[14]. Moreover, the separated MM components may also provide additional biomarkers for various disease applications [13], [15]–[17].

A number of methods have been proposed to separate the metabolite and MM signals in short-TE MRSI data. One approach is to suppress the metabolite or MM signals during the data acquisition stage by exploiting their longitudinal relaxation (*T*_1_) or diffusion property differences [18]. Examples include the most commonly used inversion recovery (IR) based excitation strategies, which are designed to null either the metabolite (with longer *T*_1_’s) or MM (with shorter *T*_1_’s) signals to measure the other [11], [19]–[22]. Methods that use two acquisitions to obtain both metabolites and MMs have also been proposed [13], [23]. Due to the variable ranges of *T*_1_ values for different molecules in vivo, complete nulling of metabolites or MMs is impossible and additional processing are usually needed to further remove the residual spectral components. If both metabolites and MMs are desired, two acquisitions are needed which will inherently increase the imaging time.

An alternative signal processing based approach is to model the overall short-TE data using parametric models of metabolites and MMs individually. The separation is then achieved by estimating the model parameters for each component from the data, e.g., solving a constrained nonlinear least-square problem [11], [24]–[29]. Improved fitting strategies have been proposed to take advantage of the fast decaying nature of the MM signals for better separation. Specifically, one can fit and back-extrapolate the metabolites using a truncated FID with negligible MM contributions and estimate the MM component by subtracting the extrapolated metabolite fits from the original signal [27], [30], [31]. Iterative subtraction and refitting can be done for improved separation performance. However, these methods are sensitive to model mismatch and noise, and often lead to substantial voxel-to-voxel estimation variations for practical MRSI data. Nonetheless, inspired by these parametric models, we recognized that the metabolites and MMs have their distinct spectral patterns specified by just a few spectral parameters, e.g., concentrations, resonance frequencies, and lineshapes, thus should reside in their own nonlinear low-dimensional manifolds embedded in the original high-dimensional space [32]. We hypothesized that these manifolds could be learned from specially designed training data and used as effective constraints for metabolite and MM separation.

While learning nonlinear low-dimensional models from high-dimensional heterogeneous data has been a major challenge in machine learning, recent breakthroughs in deep learning have enabled excellent solutions to such problems [33]–[35]. Leveraging this progress, deep neural networks have been successfully adapted to process and quantify short-TE MRSI data with MM separation/removal capability [36]–[39]. The initial attempts have been focused on training an end-to-end network that learns the inverse function to directly map the noisy and artifact containing data to the desired spectral parameters [36], [39], spectra with MM components removed [37], or even a group of networks to extract individual metabolites [38]. These methods require the complicated networks to simultaneously capture the physical model, metabolite and MM spectral variations, and all other nuances related to noise, artifacts, and acquisition designs. Recently, an alternative approach has been proposed to use deep networks to learn a low-dimensional representation of general MR spectra and use this as a prior in a constrained reconstruction formalism instead of a direct inverse mapping. This approach not only simplifies the learning problem but also allows for more flexible integration with the physical forward models for different acquisition designs. And it has been successfully applied to ^31^P-MRSI reconstruction [40].

Inspired by this approach, we proposed here a novel method to separate metabolite and MM signals for short-TE ^1^H-MRSI by learning their distinct nonlinear low-dimensional models and incorporating the learned models into a constrained reconstruction formulation. Specifically, we proposed a new strategy that combines supervised and unsupervised learning to train two special deep autoencoders (DAEs) to learn efficient low-dimensional representations that are specific to metabolites and MMs, respectively. We devised a formulation to integrate the learned representations as effective constraints with a spatiospectral encoding model for joint reconstruction as well as signal separation. An efficient algorithm was developed to solve the resulting optimization problem. We demonstrated the efficient and component-specific low-dimensional representations learned by our DAEs for metabolites and MMs, respectively. Numerical simulations and in vivo experiments were performed to illustrate the superior separation performance achieved by the proposed method over the standard parametric fitting method. Theoretical convergence analysis for the proposed algorithm was also provided. The following sections describe the proposed model learning strategy, reconstruction formulation, and numerical algorithm in details.

## II. Background

### A. Metabolite and Macromolecule Signal Separation

In a short-TE ^1^H-MRSI acquisition, the data will contain non-negligible contributions from both a metabolite component *ρ*_*met*_(**r**, *t*) and a macromolecule component *ρ*_*MM*_(**r**, *t*). The goal of separating these two signal components can be mathematically defined as estimating *ρ*_*met*_(**r**, *t*) and *ρ*_*MM*_(**r**, *t*) from their summation:

(1) $$\rho (r,t)=\rho_{met}(r,t)+\rho_{MM}(r,t),$$

which is an ill-posed problem. Solving this problem requires effective constraints. One of the most common approaches is to impose parametric models on each component, i.e., *ρ*_*met*_(**r**, *t*) = *f*_*met*_(*t*; *α*(**r**)) and *ρ*_*MM*_(**r**, *t*) = *f*_*MM*_(*t*; *β*(**r**)) where ***α***(**r**) and ***β***(**r**) contain spectral parameters for the metabolites and MMs, respectively. While *f*_*met*_(*t*; *α*(**r**)) usually incorporates resonance structures/metabolite basis generated by quantum mechanical simulations or phantom measurements, models for *f*_*MM*_(*t*; *β*(**r**)) are generally considered to be less molecule-specific (some MM peaks can be attributed to specific amino-acid residues in various proteins which may help to generate potentially stronger spectral priors [19]). As a result, different models including polynomials [27], [41], splines [24], [28], wavelets [26], and Gaussian lineshapes [13], [21], [22], [29] have been considered for *f*_*MM*_. Gaussian lineshape based models with a priori determined chemical shift frequencies (from extensive in vivo and in vitro experiments) have shown a great balance between model complexity and fitting accuracy.

Specifically, a widely used model for both metabolites and MMs for an individual FID in short-TE ^1^H-MRSI data can be written as follows [14], [37], [42]

(2) $$\rho (t)=\sum_{m=1}^{M}c_{m}e^{i\left(\phi_{0}+\phi_{m}\right)}v_{m}(t)e^{-t/T_{2,m}^{*}+i2\pi \delta f_{m}t}+\sum_{l=1}^{L}b_{l}e^{i\left(\phi_{0}+\psi_{l}\right)}e^{-t^{2}\frac{\pi^{2}W_{l}^{2}}{4\text{ln}(2)}+i2\pi \delta f_{l}t},$$

where the first summation represents the metabolite signals with *c*_*m*_, $T_{2,m}^{*}$, and *δf*_*m*_ denoting the concentrations coefficients, physiology/experiment-dependent lineshapes and frequency shifts for individual molecules, respectively, and {*v*_*m*_(*t*)} corresponds to the metabolite basis. The second term captures the MM signals where *W*_*l*_ and *δf*_*l*_ denote the Gaussian linewidths and resonance frequencies for each MM group. The variables *ϕ*_0_, *ϕ*_*m*_, and *ψ*_*l*_ are a global zeroth-order phase and molecule-dependent phases. While directly estimating all these parameters from a single FID or spectrum can be rather challenging and lead to large estimation variances, these models imply that the metabolite and MM signals may reside in their own nonlinear low-dimensional manifolds, which we believe can be learned and then used as effective constraints for metabolite and MM separation.

## III. Proposed Method

### A. Learning Component-Specific Low-Dimensional Models

**Fig. 1.**
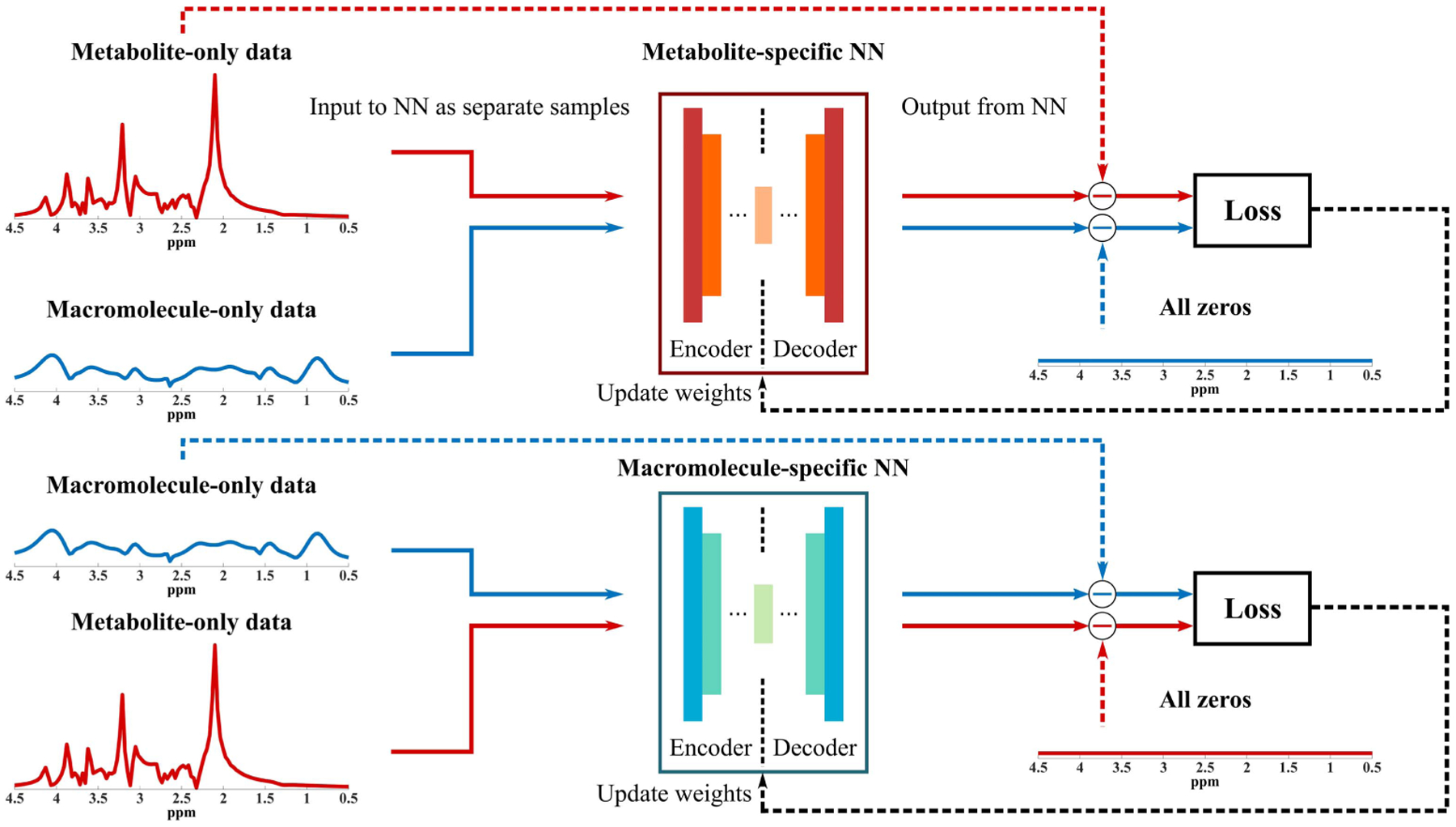
Illustration of the proposed model learning strategy with a mixture of supervised and unsupervised learning. Specifically, two DAEs are designed to capture the metabolite and MM-specific low-dimensional representations. For metabolite DAE (metabolite-specific NN), the unsupervised part enforces the network to extract a set of low-dimensional features that can approximate the metabolite signals accurately while the supervised part uses zeros as labels for the corresponding MM inputs. This enforces the network to learn to minimize its representation power of the MM signals, which will be useful for signal separation. A similar training strategy is applied to MM-specific DAE (macromolecule-specific NN) with the roles of metabolites and MMs exchanged. The mathematical formulations of this training strategy are provided in Eqs. (3) and (4).

Learning a single low-dimensional model for MR spectra by treating the entire spectrum as a point in a high-dimensional space has been investigated in [40], [43], [44]. While these learned models are powerful for spatiospectral reconstruction or denoising, they are not suited to address the signal separation problem, which requires component-specific constraints. The metabolite and MM signals have their own unique signal characteristics (e.g., distinct spectral features and parameter distributions); thus, separate models can be learned to capture the low-dimensional manifolds where they reside on or close to. However, a straightforward application of the previously described DAE [40] trained using metabolites and MMs individually may not be effective since they are not optimized to differentiate the two spectrally overlapping signal components. Therefore, we proposed here a mixed supervised and unsupervised learning strategy to address this issue. Specifically, we seek to train a DAE that can extract an accurate and efficient low-dimensional representation of the metabolite (or MM) signals with simultaneously minimal representation capability of the other component. Figure 1 provides a graphical illustration of this special model learning strategy. Mathematically, the learning problems are formulated as follows, for metabolites:

(3) $$\left\{{\hat{\theta }}_{met}\right\}=\text{arg}\;\underset{\theta_{\text{met }}}{\text{min}}\frac{1}{N}\left[\sum_{n=1}^{N}\epsilon \left(x_{\text{met }}^{n}-N_{\text{met }}\left(x_{\text{met }}^{n};\theta_{\text{met }}\right)\right)+{\left\|N_{met}\left(x_{mm}^{n};\theta_{met}\right)\right\|}_{2}^{2}\right],$$

and for macromolecules:

(4) $$\left\{{\hat{\theta }}_{mm}\right\}=\text{arg}\;\underset{\theta_{mm}}{\text{min}}\frac{1}{N}\left[\sum_{n=1}^{N}\epsilon \left(x_{mm}^{n}-N_{mm}\left(x_{mm}^{n};\theta_{mm}\right)\right)+{\left\|N_{mm}\left(x_{met}^{n};\theta_{mm}\right)\right\|}_{2}^{2}\right]$$

${\left\{x_{met}^{n}\right\}}_{n=1}^{N}$ and ${\left\{x_{mm}^{n}\right\}}_{n=1}^{N}$ are training sets (FIDs) for the metabolites and MMs, respectively, with each $x^{n}\in R^{2T}$ being a sample with real and imaginary parts concatenated. *N* is the number of training samples and *T* is the length of the FID. $N_{met}(.,.)$ and $N_{mm}(.,.)$ denote the metabolite and MM component-specific neural networks (NNs) parameterized by ***θ***_*met*_ and ***θ***_*mm*_, respectively. Here in the first term of Eq. (3), *ϵ* represents the loss for training that measures the error between the original metabolite input and the network approximation. This term enforces the NN to learn an accurate low-dimensional representation of the metabolite signals. The second term of Eq. (3), appearing as a regularization, is designed to minimize the output of the metabolite NN that corresponds to the MM data $\left(x_{mm}^{n}\right)$. This can also be viewed as using zeros as the labels for the MM input. As a result, the metabolite NN is trained to learn a representation specific to metabolite signal features with minimal representation power for MMs. Likewise, the two terms of Eq. (4) serve a similar purpose (to capture MM-specific low-dimensional features while minimizing metabolite representation). We hypothesized that DAEs trained separately in this fashion would have not only the ability to extract accurate nonlinear low-dimensional representations of metabolites and MMs individually but also the desired property that inaccurately models the other component. The specific network has an embedded “bottleneck” encoding-decoding structure that encodes the high-dimensional data into a set of *L*-dimensional features that can recover the original data, where *L* is referred to as the model order below. More details on the network are provided in the supplementary materials (Fig. S4).

### B. Signal Separation Using the Learned Models

With the trained DAEs $N_{met}(.,.)$ and $N_{mm}(.,.)$ capturing learned metabolite and MM-specific models, one remaining challenge is to effectively utilize the learned models for signal separation from practical ^1^H-MRSI data. To this end, we proposed a regularized reconstruction formulation that integrates the forward spatiospectral encoding model with *B*_0_ inhomogeneity correction capability and the two learned models for metabolite and MM separated reconstruction. Specifically, we formulated the separation problem as:

(5) $$\underset{X_{met}\in X_{1},X_{mm}\in X_{2}}{\text{min}}{\left\|d-\Omega \left\{FB⊙\left(X_{met}+X_{mm}\right)\right\}\right\|}_{2}^{2}+\lambda_{1}\sum_{n=1}^{N}{\left\|N_{met}\left(X_{met}^{n}\right)-X_{met}^{n}\right\|}_{2}^{2}+\lambda_{2}\sum_{n=1}^{N}{\left\|N_{mm}\left(X_{mm}^{n}\right)-X_{mm}^{n}\right\|}_{2}^{2}+\lambda_{3}{\left\|D_{w}\left(X_{met}+X_{mm}\right)\right\|}_{F}^{2},$$

where $X_{met}\in C^{N\times T}$ and $X_{mm}\in C^{N\times T}$ are matrix representations of the spatiotemporal functions of interest for the metabolite and MM components, with each row being a *T*-point FID and *N* the number of voxels. The feasible sets $X_{1}$, $X_{2}$ are balls with large enough radius that contain ground-truth representations.^1^ **B** models the linear phases induced by *B*_0_ inhomogeneity, ⊙ represents a point-wise multiplication, **F** denotes the Fourier transform, **Ω** is a (*k, t*)-space sampling operator (allows for flexible sampling designs), and **d** is a vector containing the noisy measured data. The first term enforces the imaging model and data consistency. The next two terms impose the priors that FID signals of metabolites and MMs belong to their own low-dimensional manifolds captured by the learned DAEs. The last term is a spatial smoothness constraint with **D**_*w*_ being a weighted finite-difference operator [45], and ‖·‖_*F*_ denoting the Frobenius norm. Eq. (5) results in a high-dimensional optimization problem, which is challenging to solve due to the presence of both nonlinear functions related to the DAEs and quadratic functions of **X**_*met*_ and **X**_*mm*_.

### C. Optimization Algorithm

We developed an efficient algorithm to address the computational challenges associated with the problem in Eq. (5). Specifically, we introduced an auxiliary variable

(6) $$S=B⊙\left(X_{met}+X_{mm}\right)$$

and reformulated the problem as:

(7) $$\begin{array}{l} \left\{{\hat{X}}_{met},{\hat{X}}_{mm},\hat{S}\right\}=\text{arg}\;\underset{X_{met},X_{mm},S}{\text{min}}\|d-\Omega \{FS\}{\|}_{2}^{2}+\lambda_{1}\sum_{n=1}^{N}{\left\|N_{met}\left(X_{met}^{n}\right)-X_{met}^{n}\right\|}_{2}^{2}+\lambda_{2}\sum_{n=1}^{N}{\left\|N_{mm}\left(X_{mm}^{n}\right)-X_{mm}^{n}\right\|}_{2}^{2}+\lambda_{3}{\left\|D_{w}\bar{B}⊙S\right\|}_{F}^{2}\;\\ s.t.\;B⊙\left(X_{met}+X_{mm}\right)=S,X_{met}\in X_{1},\;X_{mm}\in X_{2}, \end{array}$$

where $\bar{B}$ denotes element-wise conjugate of **B**. Then, the alternating direction method of multipliers (ADMM) was adapted to solve this equivalent problem [46], in which it was decomposed into simpler linear least-squares problems and nonlinear problems that can be solved in a parallel fashion. More specifically, the following subproblems were solved iteratively:

- Update **X**_*met*_ with fixed $X_{mm}^{\left(i\right)}$ and **S**^(*i*)^ as follows (*i* is the iteration index)
  (8) $$X_{met}^{\left(i+1\right)}=\text{arg}\;\underset{X_{met}\in X_{1}}{\text{min}}\lambda_{1}\sum_{n=1}^{N}{\left\|N_{met}\left(X_{met}^{n}\right)-X_{met}^{n}\right\|}_{2}^{2}+\frac{\rho }{2}{\left\|B⊙\left(X_{met}+X_{mm}^{\left(i\right)}\right)-S^{\left(i\right)}+\frac{Y^{\left(i\right)}}{\rho }\right\|}_{F}^{2}$$
  where **Y**^(*i*)^ is the Lagrangian multiplier and *ρ* is the penalty parameter.
- Update **X**_*mm*_ with fixed $X_{met}^{\left(i+1\right)}$ and **S**^(*i*)^ as
  (9) $$X_{mm}^{\left(i+1\right)}=\text{arg}\;\underset{X_{mm}\in X_{2}}{\text{min}}\lambda_{2}\sum_{n=1}^{N}{\left\|N_{mm}\left(X_{mm}^{n}\right)-X_{mm}^{n}\right\|}_{2}^{2}+\frac{\rho }{2}{\left\|B⊙\left(X_{met}^{\left(i+1\right)}+X_{mm}\right)-S^{\left(i\right)}+\frac{Y^{\left(i\right)}}{\rho }\right\|}_{F}^{2}$$
- Update **S** with $X^{\left(i+1\right)}=X_{met}^{\left(i+1\right)}+X_{mm}^{\left(i+1\right)}$ by solving
  (10) $$S^{\left(i+1\right)}=\text{arg}\;\underset{S}{\text{min}}\|d-\Omega \{FS\}{\|}_{2}^{2}+\lambda_{3}{\left\|D_{w}\bar{B}⊙S\right\|}_{F}^{2}+\frac{\rho }{2}{\left\|B⊙X^{\left(i+1\right)}-S+\frac{Y^{\left(i\right)}}{\rho }\right\|}_{F}^{2}$$
- Update **Y** as
  (11) $$Y^{\left(i+1\right)}=Y^{\left(i\right)}+\rho \left(B⊙X^{\left(i+1\right)}-S^{\left(i+1\right)}\right)$$

Subproblems (I) and (II) contain both terms associated with the nonlinear networks, $N_{met}(.,.)$ and $N_{mm}(.,.)$, and can be solved using a generic nonlinear optimization solver. Subproblem (III) is a typical linear least-squares problem with a quadratic regularization. Note that although directly minimizing Eq. (8) and Eq. (9) are very high-dimensional problems for which computing the gradient is very demanding, it can be solved in a voxel-by-voxel fashion since the Frobenius norm term is separable for all the voxels (i.e., individual rows in **X**). Based on the autoencoder design, the gradients for individual voxels can be efficiently calculated through backpropagation. More specifically, denote

(12) $$f_{n}\left(X_{met}^{n},X_{mm}^{n}\right)=\lambda_{1}{\left\|N_{met}\left(X_{met}^{n}\right)-X_{met}^{n}\right\|}_{2}^{2}+\lambda_{2}{\left\|N_{mm}\left(X_{mm}^{n}\right)-X_{mm}^{n}\right\|}_{2}^{2}+\frac{\rho }{2}{\left\|{\left[B⊙X-S^{\left(i\right)}+\frac{Y^{\left(i\right)}}{\rho }\right]}_{n}\right\|}_{2}^{2}$$

as the cost function for the *n*th voxel, the then gradients for the metabolite component can be written as:

(13) $$\nabla f_{n}\left(X_{met}^{n}\right)=2\lambda_{1}{\left(J_{N_{met}}-I\right)}^{T}\left(N_{met}\left(X_{met}^{n}\right)-X_{met}^{n}\right)+\rho B_{\left(n\right)}^{H}\left(B_{\left(n\right)}X_{n}-S_{n}^{\left(i\right)}+\frac{Y_{n}^{\left(i\right)}}{\rho }\right)$$

where $J_{N_{met}}\in R^{T\times T}$ is the Jacobian of the metabolite network, **I** is a *T* × *T* identity matrix, and **B**_(*n*)_ represents a diagonal matrix formed by the *n*th row of **B**. The gradients for MM component can be derived similarly (omitted due to space constraint). And the Jacobians $J_{N_{met}}$ and $J_{N_{mm}}$ can be calculated through backpropagation described in [40].

Subproblem (III) is equivalent to solving a system of linear equations with a spatial regularization on the overall spatiotemporal function (due to the way the auxiliary variable is introduced). The iteration is terminated until a specified iteration number is reached (e.g., 20) or the relative change between $X_{met}^{\left(i\right)}$ and $X_{met}^{\left(i+1\right)}$ is below a threshold (e.g., 10^−4^).

### D. Training Data Generation

One common issue of training deep neural networks is the requirement of a large number of high-quality training data. Strategies that combine spectral fitting models, quantum mechanical (QM) simulations and experimental data have been described in several literature to address this issue for spectral model learning [37], [40], [47]. Utilizing a similar strategy here, we generated metabolite and MM training data separately using the model in Eq. (2). For metabolites, the basis **v**_*m*_(*t*) were generated from QM simulations using the NMRScopeB software [48], for both the FID (pulse and acquire) and semi-LASER excitation schemes with different TEs [7]. These molecule specific resonance structures can be assumed to be invariant with respect to different subjects. Meanwhile, the empirical distributions of the spectral parameters, i.e., *c*_*m*_, $T_{2,m}^{*}$, and *δf*_*m*_ were estimated from literature values as well as fitting experimental high-SNR, low-resolution MRSI data from healthy volunteers [37], [43], [47]. The empirical distributions were fitted to parametric Gaussian distributions to allow for generating more randomly distributed parameters. The global zeroth-order phase was generated from a Gaussian distribution with mean zero and standard deviation of 25 degrees, and Gaussian distributed molecule dependent phases were also introduced to simulate more realistic signal variations (with mean zero and standard deviation of 10 degrees). Finally, the metabolite basis and parameters randomly sampled from these distributions were combined using the model in Eq. (2) to generate 300,000 ^1^H MR spectra. Metabolites commonly observed and quantified in ^1^H-MRSI experiments are considered, i.e., N-acetylaspartate (NAA), creatine (Cr), choline (Cho), glutamate (Glu), glutamine (Gln), myo-inositol (mI), gamma-Aminobutyric acid (GABA), glutathione (GSH) and lactate (Lac). For MMs, another 300,000 training samples were generated using a similar procedure and the model in Eq. (2). 13 commonly reported MM resonances with mean *δf*_*l*_’s equal to 0.9, 1.21, 1.38, 1.63, 2.01, 2.09, 2.25, 2.61, 2.96, 3.11, 3.67, 3.8, and 3.96 ppm were included. The parameters *b*_*l*_ (concentration coefficients) and *W*_*l*_ (linewidths) for different peak groups were assumed to follow Gaussian distributions. The mean values were acquired from [37] with standard deviations specified as 20% of the means to introduce relative peak variations. A global scaling factor was introduced to the MM coefficients to reflect experimentally observed metabolite-to-MM signal ratios. Finally, all data were normalized to the range of −1 to 1 for training.

### E. Other Implementation Details

Among the 300,000 training data, 200,000 were used for training and 100,000 for testing. All the model parameters were upper and lower bounded based on our own and other published ^1^H-MRSI data [37], [38]. Specifically, the $T_{2,m}^{*}$ values were lower bounded by 5 ms and upper bounded by 200 ms, and the *c*_*m*_ values were bounded between 0 and 2 with the mean NAA concentration being 1. The linewidth *W*_*l*_ values were bounded within the range of 5 to 70 Hz, and *b*_*l*_’s were lower bounded by 0. The parameters were first generated, and the values outside these ranges were excluded. The spectral bandwidth (BW) was fixed at 2000 Hz. Similar to the DAE used in [40], the metabolite and MM networks have a fully-connected structure of 2*T* − 1000 − 250 − 100 − *L* − 100 − 250 − 1000 − 2*T*. Both Tanh and ReLu units can be used in the nonlinear hidden layers except the middle linear layer (with similar performances). The results shown below were from ReLu. The learned network models were first evaluated with a range of *L*’s. The exact *L* for phantom and in vivo data processing was chosen, such that the NNs for metabolites and MMs have similar approximation errors (around 5%). Note that the training only needs to be performed once for a fixed excitation scheme (with specific choices of RF pulses, TE, and field strength). All the networks were implemented in PyTorch and trained using an NVIDIA RTX Titan graphics processing unit on Windows 10 using the Adam optimizer [49] with a batch size of 500, an initial learning rate of 0.001, and 300 epochs while the other parameters remained as default. The Broyden–Fletcher–Goldfarb–Shanno (BFGS) algorithm was used to solve the optimization problem for individual voxels in Eqs. (8) and (9), and the linear conjugate gradient was used to solve Eq. (10) [50].

## IV. Simulation and Experimental Settings

### A. Numerical Simulations

The component-specific representation power of our learned models was first evaluated. Specifically, we validated the approximation accuracy of the trained DAE-based metabolite and MM-specific low-dimensional models with comparison to linear subspace models (estimated from the same training data) [43], at different model orders. Testing metabolite and MM data were generated and passed through the trained metabolite and MM-specific networks, respectively. The errors of the same test data projected onto the metabolite and MM subspaces were also calculated. More specifically, two Casorati matrices were first constructed by stacking all the training data for metabolites or MMs, respectively. Then the component-specific subspace was obtained by SVD with a rank truncation (*L*, the model order). Finally, the testing data were projected onto the two subspaces separately to evaluate approximation accuracy [40], [43]. The approximation performance was evaluated quantitatively using a relative *ℓ*_2_ error defined as:

(14) $$\text{err}=\frac{{\left\|X_{\text{true}}-\hat{X}\right\|}_{F}}{{\left\|X_{\text{true}}\right\|}_{F}}$$

where **X**_*true*_ denotes the original data (each column being an FID), and $\hat{X}$ represents the model approximation or reconstructed data (see below).

A numerical phantom was constructed to evaluate the signal separation performance using the learned DAE-based nonlinear models (details of the phantom generation process can be found in the supplementary materials). In short, brain tissue fraction maps for gray matter (GM), white matter (WM), and cerebrospinal fluid (CSF) were first obtained from an in vivo anatomical *T*_1_-weighted image. Then regional spectral parameters for different ^1^H metabolites and MM components described in the Training Data Generation section were assigned based on literature values [14], [37], [51]. The constant parameters in each region were subsequently combined using tissue fraction maps as weightings to simulate continuously varying parameters across the brain. Finally, the parameters at different voxels along with metabolite basis {*v*_*m*_} were fed into Eq. (2) to synthesize spatially localized FIDs. To simulate a more realistic scenario, voxel-dependent random frequency shifts (mean zero and standard deviation of 5 Hz) for different molecules, as well as *B*_0_ inhomogeneity (mean zero and standard deviation of 10 Hz), were also introduced. A lesion-like feature with significantly altered metabolite ratios (e.g., a factor of three higher Cho and lower concentrations for other metabolites) and a higher MM level was included. Noisy data were generated by adding complex white Gaussian noise with different SNRs to the simulated (*x, t*)-space data. The SNR is defined with respect to the maximum NAA peak amplitude within the FOV. After the proposed reconstruction, the separated metabolite and MM components were fitted individually using a metabolite-only and a MM-only parametric model from Eq. (2) in a voxel-by-voxel fashion to produce molecular maps. In comparison, a direct parametric fitting was also performed to the original data without the proposed separation. An FID truncation was performed to fit the metabolites first which were then back-extrapolated for subtraction to fit the MMs. A metabolite refitting was done after subtracting the fitted MMs from the original data. All fittings were done using in-house implementations which have been validated against the time-domain fitting using the jMRUI package [27], [48] (The customized implementations provided more flexibility for further optimizations).

### B. In Vivo Experiments

We have evaluated the performance of the proposed method using practical in vivo data acquired from five healthy volunteers with approval from the local Institutional Review Board. Experimental brain MRSI data were acquired on a 3T Prisma scanner equipped with a 20-channel head coil using both an FID-MRSI (pulse and acquire) sequence and a semi-LASER MRSI short-TE sequence (sLASER). We chose these two sequences because they are among the most commonly used short-TE data acquisition schemes and require rather different metabolite basis sets which serves to demonstrate that the proposed method can be flexibly adapted to work with any sequences. The parameters for the FID-MRSI sequence were as follows: TR/TE = 800/4 ms, field-of-view (FOV) = 230 × 230 mm^2^, slice thickness = 10 mm, matrix size = 36 × 36, spectral bandwidth (BW) = 2000 Hz and 512 FID samples. The total acquisition time was about 13.5 minutes with elliptical sampling. The parameters for the sLASER sequence were: TR/TE = 1600/40 ms, FOV = 180×190 mm^2^, slice thickness = 15 mm, matrix size = 24×24, 2000 Hz BW and 1024 FID samples. The total acquisition time was about 16 minutes. A 60 Hz weak water suppression and carefully placed outer volume suppression bands were used for all the scans. Before reconstruction, the nuisance water and lipid signals were first removed using the method in [52] followed by coil combination of the water/lipid-removed data.

## V. Results

### A. Simulation Results

**Fig. 2.**
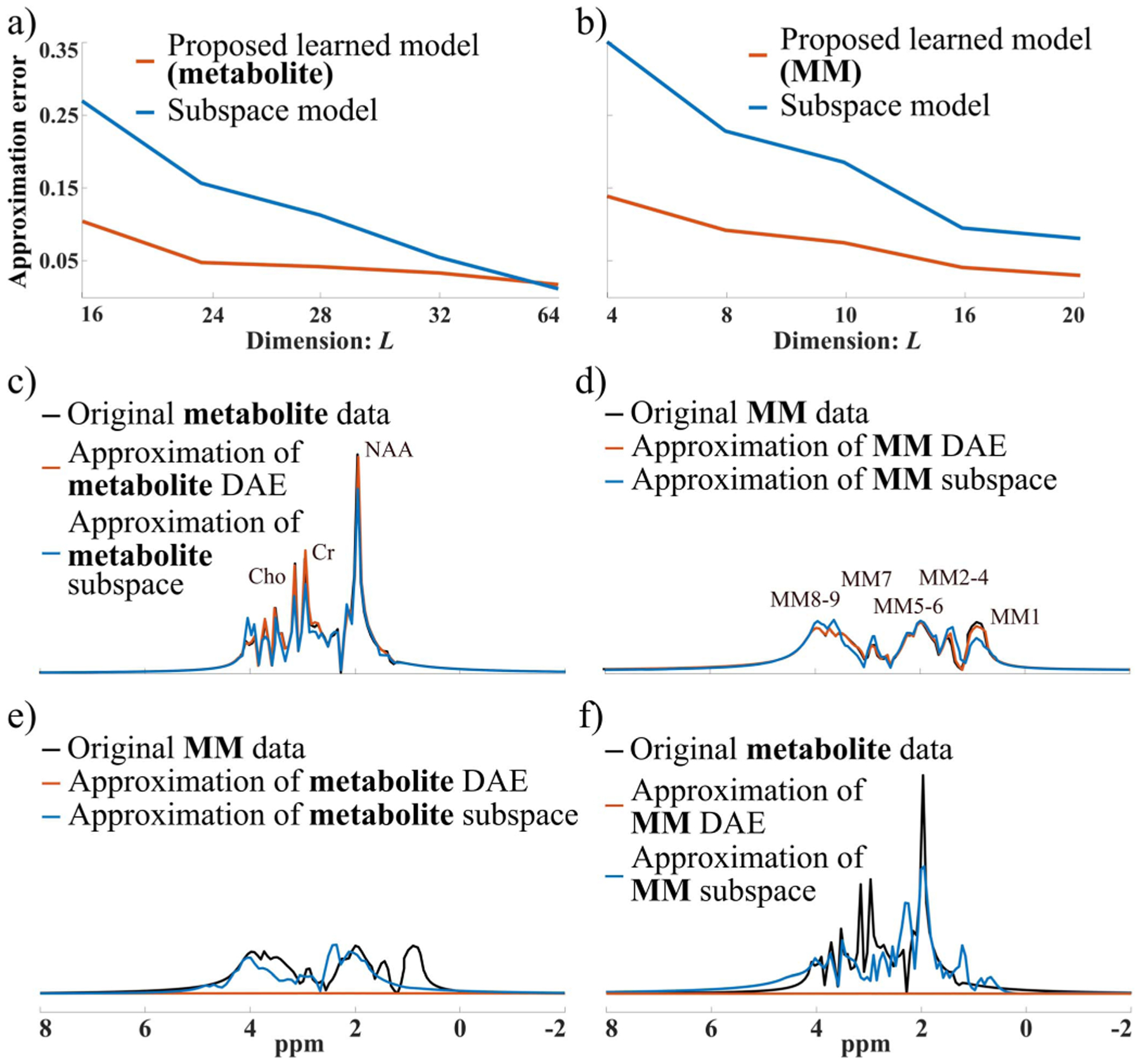
Representation capability of the learned nonlinear models: a) Approximation errors (relative *ℓ*_2_) of the trained metabolite DAE (orange curve) compared to a linear subspace model (blue curve) for the metabolite data at different *L*’s; b) Approximation errors of the MM DAE (orange curve) with comparison to a linear subspace model (blue curve); c) and e) Representative metabolite and MM spectra (black), and the approximations of each signal by the metabolite DAE (orange) and metabolite linear subspace (blue) both with *L* = 24; d) and f) Representative MM and metabolite spectra (black), and the approximations by the MM DAE (orange) and subspace (blue) with *L* = 8. It is evident that the learned DAEs have more accurate and component-specific representation than the linear subspaces.

Figure 2 compares the representation powers of our learned component-specific nonlinear low-dimensional models and the linear subspace models. As shown, the learned metabolite (Fig. 2a) and MM (Fig. 2b) DAE-based models achieved higher accuracy with lower relative *ℓ*_2_ errors than the subspace models for their respective components. With the same model order (*L*), the MM DAE has higher accuracy than the metabolite DAE. The approximations for two representative testing metabolite and MM spectra are also shown in Fig. 2c–f to further demonstrate the accuracy and specificity of the learned models. With a fixed model order *L* = 24 for metabolite and *L* = 8 for MM, the metabolite DAE can accurately capture the metabolite spectral features (Fig. 2c), exhibiting a higher accuracy than the subspace model with the same dimension. Similar results can be observed for the MM test spectrum (Fig. 2d). More importantly, the metabolite DAE offers a poor approximation of the MM spectrum as we designed it to, while the linear metabolite subspace can still capture a decent amount of MM spectral energy (Fig. 2e), implying a weaker capability for signal separation. This component-specific representation can also be observed for the MM DAE (Fig. 2f), which does not capture the metabolite spectral features, while the MM subspace can again capture a large portion of metabolite signal energy. These validate the desirable component-specific representation capability of the learned models and imply their unique potential for improved metabolite and MM separation.

**Fig. 3.**
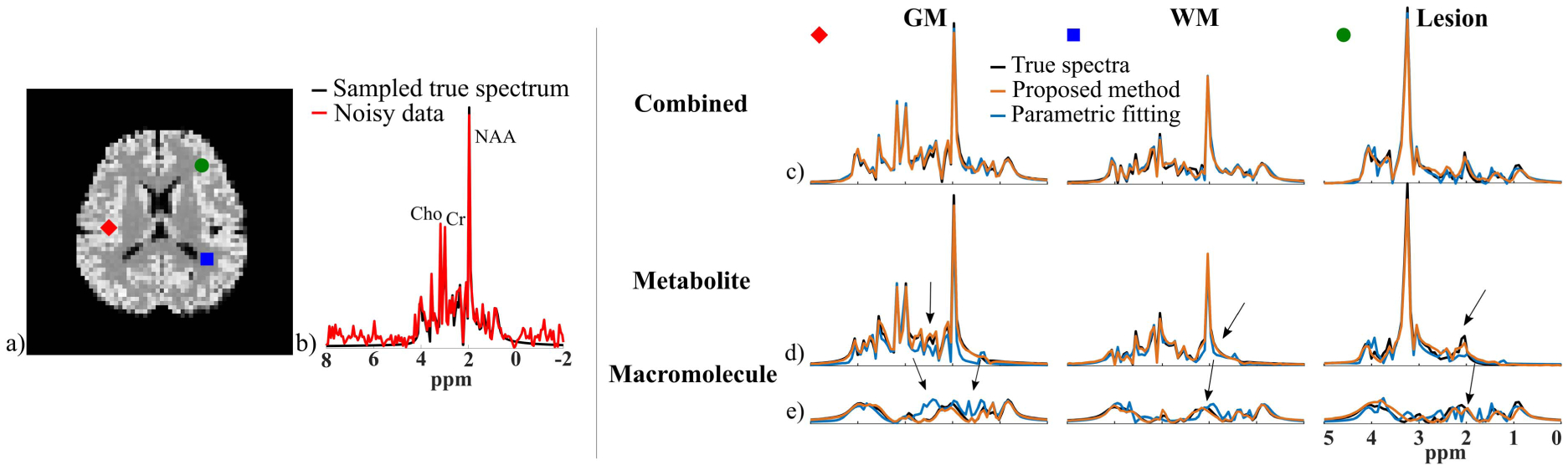
Simulation results: a) Spatial variations of the overall metabolite and MM signals in the phantom (*ℓ*_2_ integral along the FID dimension); b) A sampled voxel spectrum and its noisy counterpart (SNR = 30); c)-e) Separation results from the proposed method (orange curves) and the direct parametric fitting (blue curves, without the proposed separation) for three different voxels (in GM, WM, and Lesion). The voxel locations are indicated by different shapes in (a). Similar overall spectra (c) were produced by both methods. But the proposed method yielded more accurate separated metabolite (d) and MM (e) components. The black arrows identify some spectral features better recovered by the proposed method.

**Fig. 4.**
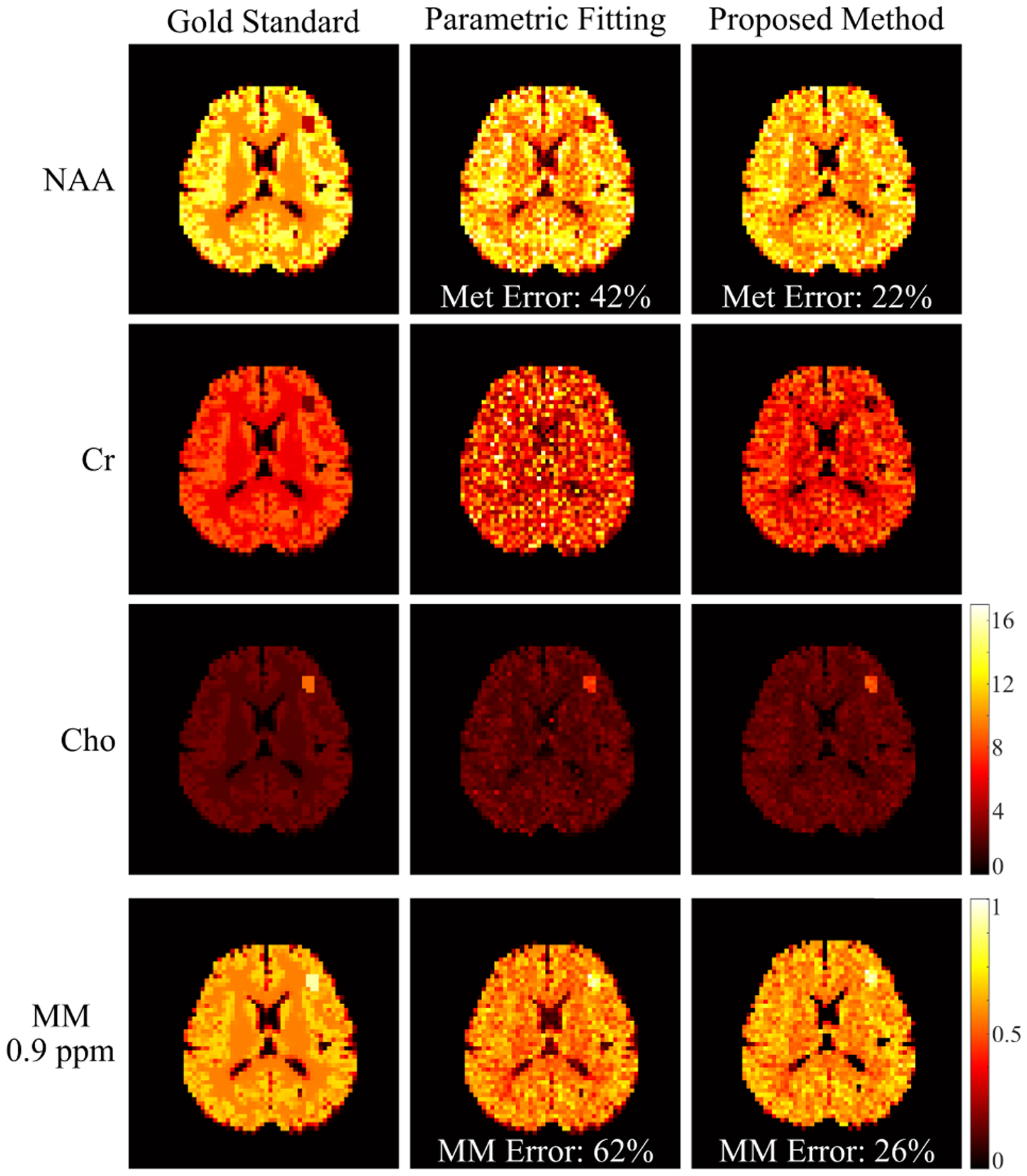
Simulation results: molecular maps of NAA, Cr, Cho, and MM from the ground truth (Gold Standard, column 1), the direct parametric fitting method (column 2) and the proposed method (column 3) are compared. For the proposed method, the maps were obtained by fitting the separated metabolite and MM components individually. The first MM peak group (located at ~0.9 ppm) is shown [37]. Note that the MM maps were normalized separately, thus having a different scaling compared to the metabolite maps. Relative *ℓ*_2_ errors for the separated metabolite and MM signals are also calculated (shown in the images). The improved signal separation offered by the proposed method lead to significantly improved molecular quantification.

A set of metabolite and MM signal separation results from the numerical phantom (SNR = 30) are shown in Figs. 3 and 4. Here the resulting spectra were shown in magnitude for visualization purpose (the real parts of reconstructed spectra can be found in the supplementary materials Fig. S1). The model order (*L*) was chosen as 24 for the metabolite DAE and 16 for the MM DAE with similar approximation errors (~5% error), which achieved a good balance between model complexity and approximation accuracy. The regularization parameters *λ*_1_ and *λ*_2_ were chosen based on a single voxel separation performance, and *λ*_3_ was chosen using the discrepancy principle and then fine-tuned by minimizing the *ℓ*_2_ errors of the final spatiospectral reconstructions. A time-domain direct parametric model-based fitting with back-extrapolation was also performed as described above, and the results were compared. The separated metabolite and MM spatiotemporal distributions from the proposed method were subject to parametric fitting (using the metabolite-only and MM-only parametric models, respectively). As can be seen, both the parametric fitting and the proposed method achieved similar estimates of the overall spectra (Fig. 3c), but the proposed method produced significantly more accurate separated metabolite and MM components (Figs. 3d and e). The metabolite maps obtained by direct parametric fitting of the overall data (Parametric Fitting) and the proposed method (separate fitting) are compared in Fig. 4. The results demonstrate the benefits of the proposed signal separation. More specifically, the metabolite maps from fitting the separated signals exhibit significantly less spatially dependent estimation variances and higher accuracy than those produced by a direct parametric fitting of the combined signals. An additional set of results from less noisy data (SNR = 60) are shown in the supplementary materials (Fig. S3).

### B. In Vivo Results

**Fig. 5.**
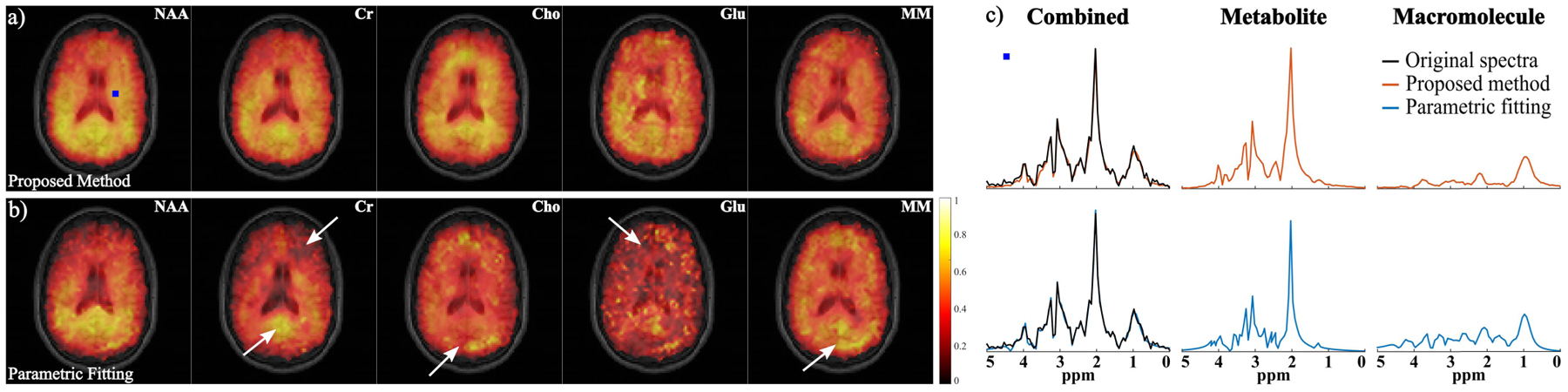
Experimental results from the in vivo FID-MRSI data: a) and b) Maps of NAA, Cr, Cho, Glu and MM estimated from the separated signals produced by the proposed method (a) and from the direct parametric fitting method (b). The molecular maps are overlaid on an anatomical image for the matched slice; c) Spatially-resolved spectra from the voxel marked by the blue symbol, with the first and second rows showing the results from the proposed method (orange curves) and parametric fitting (blue curves), respectively. The original spectra are shown in black. The overall reconstruction, as well as the separated metabolite and MM spectra, are compared. The white arrows indicate some artifact-like features generated by the direct parametric fitting which are not present in the maps produced by the proposed method.

**Fig. 6.**
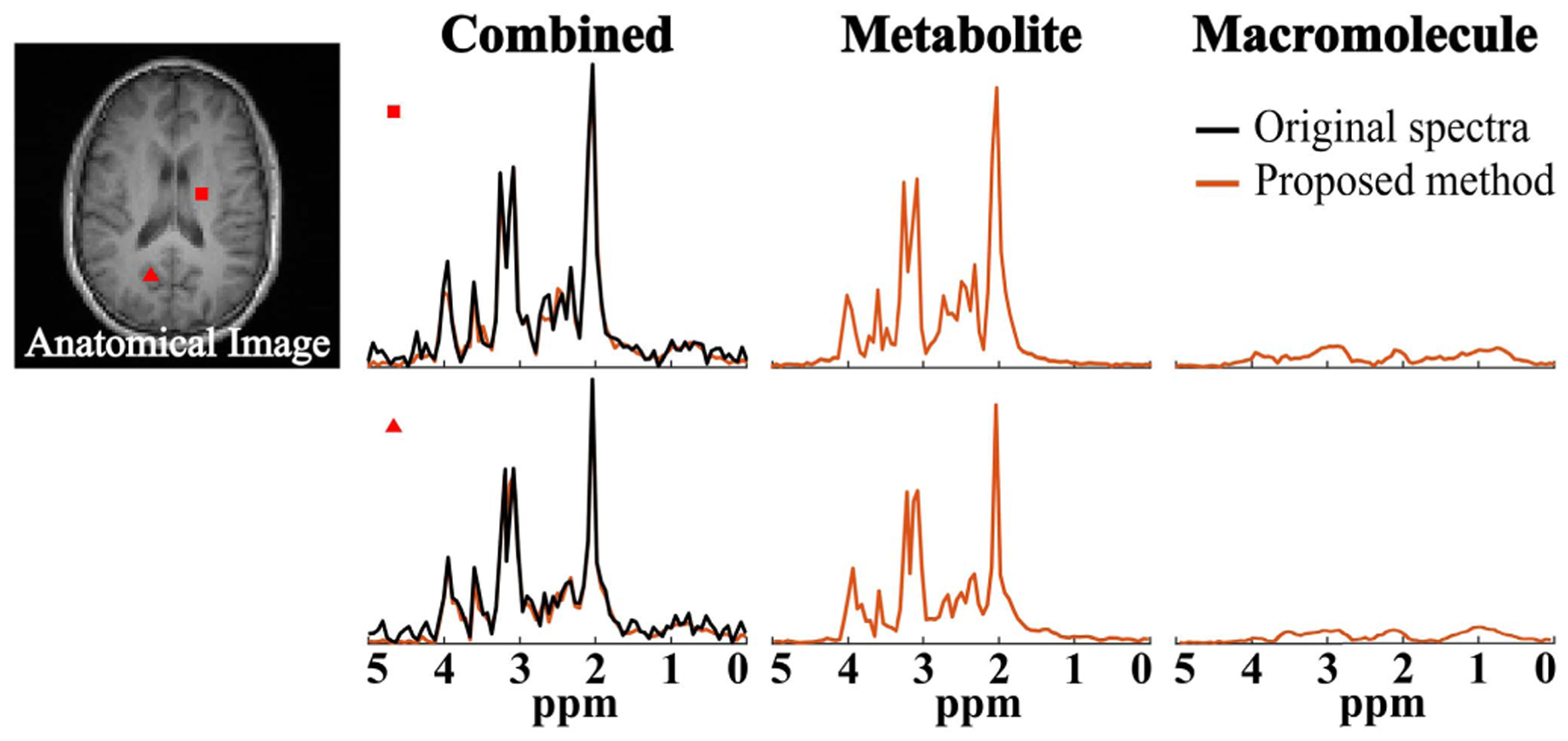
Results from the same data in Fig. 5 but with the first 36 FID points truncated (~18 ms) and zero-padded to the original length. Two representative spatially-resolved spectra from the locations marked by the corresponding symbols are shown, including the original spectra (black), the overall reconstruction, and the separated metabolite and MM spectra (orange). Significantly reduced MM signals were observed, indicating that the proposed method is not overfitting.

A set of spatially-resolved spectral reconstruction obtained by the proposed method from the in vivo FID-MRSI data are shown in Fig. 5. As can be seen, the proposed method was able to separate the metabolite and MM spectral components with a similar overall spectrum to the direct parametric fitting method. Furthermore, the metabolite and MM maps from the proposed method exhibited a higher quality with less spatial estimation variances. More specifically, the molecule maps produced by the direct parametric fitting had apparent artifacts, e.g., locally dark/bright areas and sudden discontinuities (indicated by the white arrows in Fig. 5b). These artifacts were effectively reduced in the maps from the proposed method. The relative peak intensities may not appear the same as those from standard IR-based MM measurements due to different *T*_1_ weightings (effects of no IR and the shorter TR used), e. g., a strong 0.9 ppm MM peak even before separation. Hence, results from another dataset acquired with a longer TR (1500 ms) are included in the supplementary materials (Fig. S2) to further illustrate the TR effects. We have also performed a reconstruction of the same FID-MRSI data with the first 36 time points truncated (i.e., 18 ms) to evaluate the robustness of the proposed method. As shown in Fig. 6, significantly reduced MM signals were obtained, indicating that the learned model is not overfitting.

**Fig. 7.**
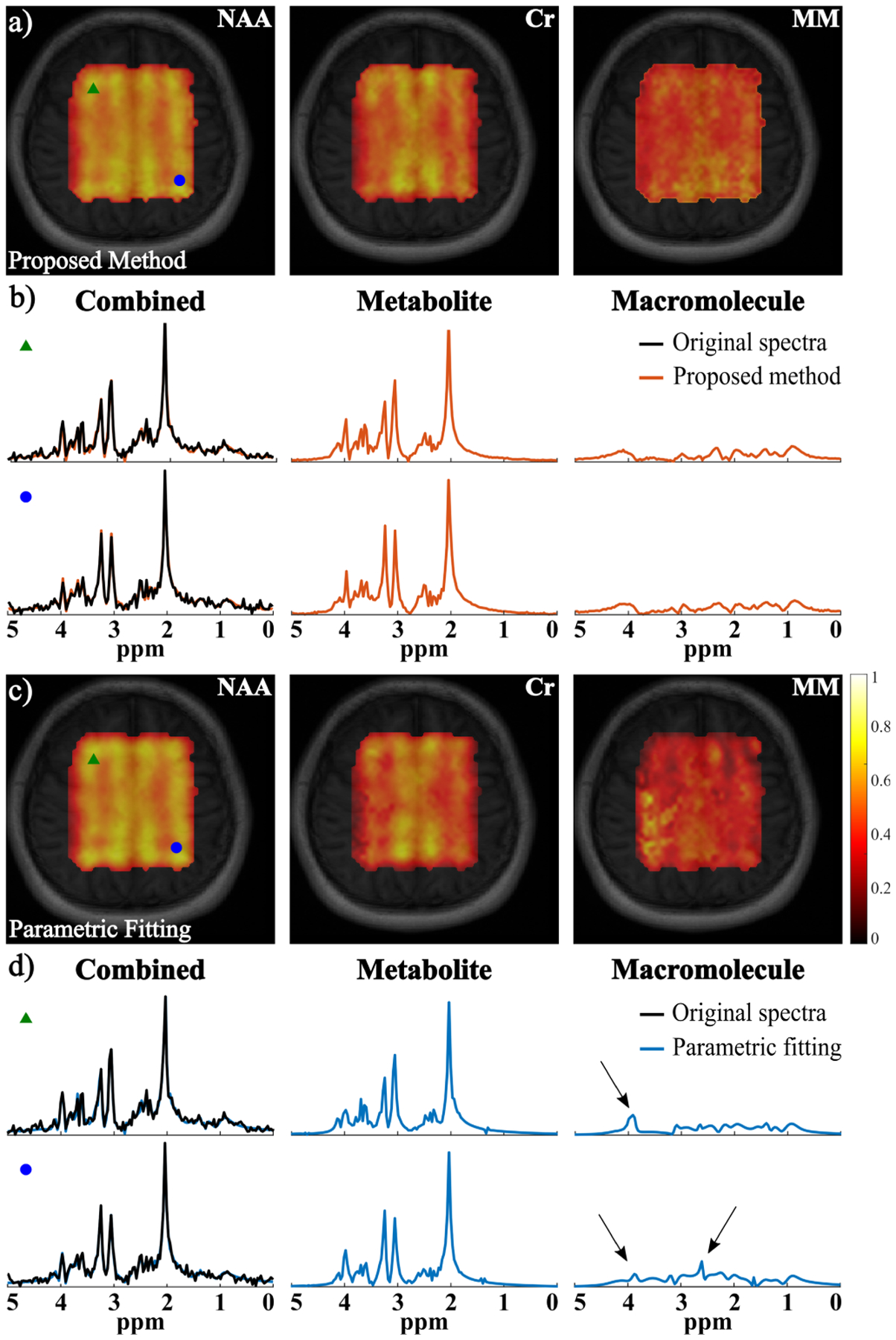
Experimental results from the in vivo sLASER data: a) Maps of NAA, Cr and MM from the proposed method (overlaid on anatomical images); b) Two representative spatially-resolved spectra (voxel locations marked by the corresponding symbols) with the original spectra (black, column 1), the overall reconstruction (column 1), and the separated metabolite (column 2) and MM (column 3) spectra; c) and d) The corresponding results from the parametric fitting method with the same arrangement. The spectra from the two methods (b and d) are from the same voxels. The arrows indicate some over and underestimation of metabolite or MM components from the parametric fitting that were mitigated by the proposed method.

Figure 7 shows the spatially-resolved spectral reconstruction from the sLASER data to further demonstrate the utility of the proposed method. Signals can only be observed from the central region of the brain due to the volume selective excitation. The proposed method again produced visually better separation, which can be observed in the metabolite and MM maps (e.g., better gray/white matter contrast and fewer artifacts) as well as the selective voxel spectra. The proposed method effectively reduced the over and underestimation of some metabolite and MM spectral components in the parametric fitting method (Figs. 7b and d). Additional metabolite maps can be found in the supplementary materials (Fig. S5). Our approach should work for any excitation as long as the corresponding metabolite basis can be obtained for training data generation.

### C. Convergence Analysis

**Fig. 8.**
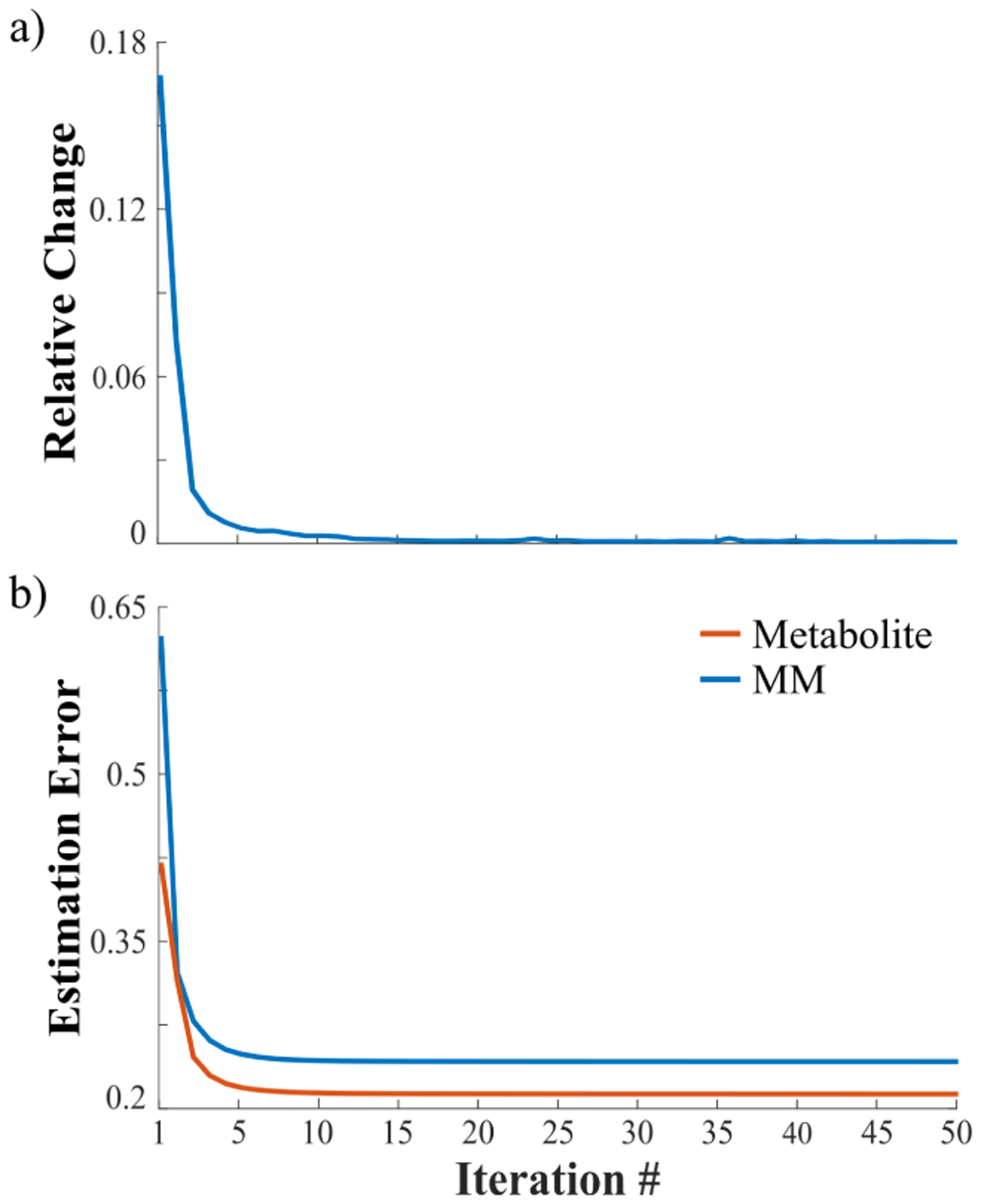
Convergence analysis of the algorithm: (a) Relative changes (in terms of *ℓ*_2_ errors) between different iterates; (b) Relative *ℓ*_2_ errors for the metabolite (orange) and MM (blue) components w.r.t. different iterations. As can be seen, the result changes minimally after 10 iterations.

We have also performed convergence analysis of the proposed algorithm. Figure 8 shows the relative changes between iterates (‖**X**^(*i*+1)^ − **X**^(*i*)^ ‖/‖**X**^(*i*)^) and relative *ℓ*_2_ errors for the metabolite and MM estimates w.r.t. the iteration number. Empirical convergence can be observed. Furthermore, our problem formulation and the ADMM-based algorithm allow us to theoretically characterize its convergence.

#### Theorem 1:

There exists a constant *ρ*_0_ such that if *ρ* ≥ *ρ*_0_, every limit point of the sequence (**X**^(*i*)^,**S**^(*i*)^) generated by the algorithm described in (8), (9), (10), and (11) is a stationary solution of the optimization problem (7) (i.e. a solution that satisfies the KKT condition).

This theorem states that for a proper choice of penalty parameter *ρ*, the sequence generated by our algorithm is guaranteed to converge to stationary points. For non-convex problems, convergence to the global minimum is often very difficult. Thus, we follow the common practice to prove a result of convergence to stationary points. We remark that even the convergence to a stationary solution is a non-trivial property because a general convergence result of ADMM for non-convex problems is still an open question. This theoretical characterization is enabled by the unique structure of our problem formulation: we show that it is a special case of the non-convex sharing problem [53], for which the convergence results have been established.

#### Proof Sketch:

If we denote **X**_1_ = vec(**X**_*met*_), **X**_2_ = vec(**X**_*mm*_), **X**_0_ = vec(**S**), then $\lambda_{1}\sum_{n=1}^{N}{\left\|N_{met}\left(X_{met}^{n}\right)-X_{met}^{n}\right\|}_{2}^{2}$ is a function of **X**_1_, which we denote as *g*_1_(**X**_1_). Similarly, $\lambda_{2}\sum_{n=1}^{N}{\left\|N_{mm}\left(X_{mm}^{n}\right)-X_{mm}^{n}\right\|}_{2}^{2}$, is a function of **X**_2_, which we denote as *g*_2_(**X**_2_). The remaining terms, ${\left\|d-\Omega \left\{FX_{0}\right\}\right\|}_{2}^{2}+\lambda_{3}{\left\|D_{w}\bar{B}⊙X_{0}\right\|}_{F}^{2}$, can be written as a function of the vectorized variable **X**_0_, which we denote as *ℓ*(**X**_0_). The constraint **B** ⊙ (**X**_*met*_ + **X**_*mm*_) = **S** can be rewritten as *A***X**_1_ + *A***X**_2_ = **X**_0_ for a certain matrix *A*. Then our optimization problem in Eq. (7) can be written in the general form of

(15) $$\begin{array}{l} \underset{X_{1}\in X_{1},X_{2}\in X_{2},X_{0}}{\text{min}}g_{1}\left(X_{1}\right)+g_{2}\left(X_{2}\right)+l\left(X_{0}\right)\\ \;s.t.\;AX_{1}+AX_{2}=X_{0}. \end{array}$$

Recognizing that this is a special case of the sharing problem in [53], we apply the convergence result provided in this work. The detailed proof is provided in the supplementary materials.

## VI. Discussion

We have successfully combined the physics-based data acquisition model with learned low-dimensional models for effective metabolite and macromolecule separation. Our unique strategy of combining supervised and unsupervised learning to discover component-specific low-dimensional manifolds is a novel attempt motivated by the nature of spectroscopic data. The proposed reconstruction represents a rigorous approach to leverage deep learning to solve this long-standing challenge. In contrast to the existing methods that learn end-to-end mappings, the proposed method allows the use of a general (*k, t*)-space sampling operator with high flexibility in the choices of sampling designs and SNR levels, and the ability to account for *B*_0_ inhomogeneity. While significant noise reduction can be observed in the separated metabolite and MM spectra due to the inherent denoising capability of the low-dimensional models used, it should be noted that the proposed method is focused on addressing the signal separation problem and not a substitute for spectral quantification. Our hypothesis is that a better separation will lead to improved spectral quantification of different components of interest, which has been supported by both simulation and experimental results. Meanwhile, we expect that the proposed method can be readily integrated with other more sophisticated parametric models for data generation and representation learning (both metabolites and MMs) as well as advanced quantification strategies for the separated signal components. This is beyond the scope of this work but will be investigated in future research.

While in this work we have only considered nine metabolites that are the common molecules of interest in most brain MRSI studies, especially at 3T, the proposed model learning and reconstruction methodologies are not limited by the number of metabolites considered. Adding more metabolites into the model will increase the model order to achieve the same approximation accuracy but will not cause substantially higher computation burden. For the other ^1^H metabolites, it will be very challenging to quantify them reliably given the SNR level and ignoring them has a minimal bias (due to their weak signals), hence we did not include them. But more metabolites can be considered when we adapt our method for data with higher SNRs from higher field strengths (e.g., 7T). One additional thing to note is that the learned MM-specific model should be able to capture potential residual lipids spectrally overlapping with the MM peaks (if sufficient lipid removal can be achieved), because of the lineshape and frequency variations introduced when generating the MM training data. Thus, small lipid residuals will not affect the model’s representation capability or metabolite separation.

One important issue with the proposed method is the choice of regularization parameters. In our current implementation for practical data, we first performed a single voxel separation for the selection of *λ*_1_ and *λ*_2_ using the parameter values from the phantom studies. The third parameter *λ*_3_ was then initialized based on the simulation studies and adjusted according to the discrepancy principle. Some minor fine-tuning together with *λ*_1_ and *λ*_2_ was performed using visual inspection of the separation reconstruction to balance SNR improvement and smoothing effects (the relative ratios between the three parameters remained the same during this step). More sophisticated parameter selection strategies can be explored in future research. Moreover, the formulation can readily be extended to incorporate other spatiospectral constraints for improved reconstruction.

The current fully-connected network-based DAE structures and the way of handling complex-valued data may be limited in scalability. We have investigated convolutional structures (with reduced numbers of training parameters) for both FID and spectral data and found that they were not as effective as our current DAEs in terms of dimensionality reduction. Various combinations of fully-connected and convolutional feature extraction layers, as well as choices of activation functions, are currently being explored. The current training data generation processes used relatively simplified spectral parameter distributions. While producing strong performance, this strategy does not fully exploit the information available from experimental ^1^H spectroscopy data. Estimation of more sophisticated distributions using such data will be studied in future work, e.g., using kernel density estimation [54].

Although Cartesian k-space sampling has been used to demonstrate the utility of the proposed method, other sampling trajectories can be considered by generalizing the forward encoding operator without having to retrain the models (another unique advantage of our approach). The most computationally expensive step in the current algorithm is solving Eq. (8) and Eq. (9) that involves backpropagation. This is, however, a highly parallelizable process that can significantly benefit from translating the current implementation to parallel computing platforms.

## VII. Conclusion

We have presented a new method to reconstruct and separate metabolite and MM signals for short-TE ^1^H-MRSI by learning the two signal components’ distinct nonlinear low-dimensional models and using the learned models as priors for reconstruction. The models were learned using two deep autoencoder based neural networks to accurately capture metabolite and MM-specific low-dimensional manifolds of their high-dimensional spectral variations. A constrained spatiospectral reconstruction formulation that exploits the learned models for signal separation was proposed and solved by an efficient ADMM-based algorithm. Significantly improved separation over the standard parametric fitting approach has been demonstrated using both simulated and experimental short-TE brain ^1^H-MRSI data. Theoretical analysis of the proposed formulation and algorithm was also provided.

## Supplementary Material

supp1-3048933

## Footnotes

1 For the empirical computation, we often set $X_{1}$, $X_{2}$ to be $C^{N\times T}$; but due to technical consideration, in the convergence results, we set them to be bounded sets.
