# Supplementary material for: Separation of Metabolites and Macromolecules for Short-TE 1H-MRSI Using Learned Component-Specific Representations
Source: IEEE Trans Med Imaging. Author manuscript; Available in PMC 2021 Apr 15. (PMC8049099; doi:10.1109/TMI.2020.3048933)
Supplement: supp1-3048933 [file NIHMS1689914-supplement-supp1-3048933.pdf]

Supplementary Materials

Proof of Theorem 1

We first restate the convergence theorem to be proved.

Theorem 1. *There exists a constant ρ_0 such that if $\rho \geq \rho_0$, every limit point of the sequence $(\mathbf{X}^{(i)}, \mathbf{S}^{(i)})$ generated by the algorithm described in (8), (9), (10), and (11) is a stationary solution of the optimization problem (7) (i.e. a solution that satisfies the KKT condition).*

We first explain more details of the transformation outlined in the proof sketch. Denote $\mathbf{X}_1 = \text{vec}(\mathbf{X}_{met})$, $\mathbf{X}_2 = \text{vec}(\mathbf{X}_{mm})$, $\mathbf{X}_0 = \text{vec}(\mathbf{S})$. Here vec is the standard vectorization operation of a matrix, i.e. write a matrix $(M_{ij})_{1\leq i\leq n_1, 1\leq j\leq n_2}$ as a vector of size $n_1 n_2$. The main purpose of this vectorization is to write the linear constraint in the standard form. Denote A as a diagonal matrix whose diagonal entries are all the entries of vec($\mathbf{B}$). Then the constraint $\mathbf{B} \odot (\mathbf{X}_{met} + \mathbf{X}_{mm}) = \mathbf{S}$ can be written as $A\mathbf{X}_1 + A\mathbf{X}_2 = \mathbf{X}_0$. To be consistent, we also need to express the objective as a function of the vectorized variables. Therefore we rewrite $\lambda_1 \sum_{n=1}^{N} \|\mathcal{N}_{met}(\mathbf{X}_{met}^n) - \mathbf{X}_{met}^n\|_2^2$ as a function of the vectorized variable $\mathbf{X}_1$, denoted as $g_1(\mathbf{X}_1)$. It is not hard to write down the closed form expression of g_1, but for the proof we do not need this expression, so we skip it. Similarly, we can rewrite $\lambda_2 \sum_{n=1}^{N} \|\mathcal{N}_{mm}(\mathbf{X}_{mm}^n) - \mathbf{X}_{mm}^n\|_2^2$, as a function of $\mathbf{X}_2$, denoted as $g_2(\mathbf{X}_2)$. We rewrite

$$\|\mathbf{d} - \Omega\{\mathbf{F}\mathbf{X}_0\}\|_2^2 + \lambda_3 \|\mathbf{D}_w \bar{\mathbf{B}} \odot \mathbf{X}_0\|_F^2, \tag{16}$$

as a function of the vectorized variable $\mathbf{X}_0$, which we denote as $\ell(\mathbf{X}_0)$.

Then our optimization problem in Eq. (7) can be written in the general form of

$$\begin{aligned} &\min_{\mathbf{X}_1 \in \mathcal{X}_1, \mathbf{X}_2 \in \mathcal{X}_2, \mathbf{X}_0} g_1(\mathbf{X}_1) + g_2(\mathbf{X}_2) + \ell(\mathbf{X}_0) \\ &s.t. \quad A\mathbf{X}_1 + A\mathbf{X}_2 = \mathbf{X}_0. \end{aligned} \tag{17}$$

This is a special case of the sharing problem [1]. And our algorithm (8), (9), (10), (11) is a special case of Algorithm 4 in [1], which converges to a stationary point under certain technical conditions described in Assumption C in [1]. Next, we verify these conditions.

Assumption C1 in [1] contains two sub-assumptions: the objective $\ell(\mathbf{X}_0)$ is Lipschitz smooth, and the constraint coefficient matrix A is full column rank (the original sub-assumption requires the coefficient matrix for each variable is full column rank; here the coefficient matrix for $\mathbf{X}_1$ and $\mathbf{X}_2$ are the same). The objective function

$$\ell(\mathbf{X}_0) = \|\mathbf{d} - \Omega\{\mathbf{F}\mathbf{X}_0\}\|_2^2 + \lambda_3 \|\mathbf{D}_w \bar{\mathbf{B}} \odot \mathbf{X}_0\|_F^2 \tag{18}$$

is a quadratic function of $\mathbf{X}_0$, thus Lipschitz smooth, i.e., there exists L such that $\|\nabla\ell(\mathbf{X}_0) - \nabla\ell(\tilde{\mathbf{X}}_0)\|_F \leq L\|\mathbf{X}_0 - \tilde{\mathbf{X}}_0\|_F$. for any $\mathbf{X}_0, \tilde{\mathbf{X}}_0$. In fact, L can be chosen as the maximum eigenvalue of the Hessian of $\ell(\mathbf{X}_0)$. This verifies the first sub-assumption. Recall that A is a diagonal matrix whose diagonal entries are all the entries of vec($\mathbf{B}$). Based on the MRSI physics, each entry of $\mathbf{B}$ is a non-zero number, thus A has full column rank. This verifies the second sub-assumption.

Assumption C2 in [1] contains two sub-assumptions: the subproblems are strongly convex with modulus $\gamma_1(\rho), \gamma_2(\rho)$ and $\gamma_0(\rho)$ respectively (these quantities may depend on ρ, thus appear in the bracket), and the penalty parameter ρ satisfies $\rho > \max\{2L^2/\gamma_0(\rho), 2L^2\}$. Since the feasible set of $\mathbf{X}_i$ is bounded, the minimum eigenvalue of the Hessian of $g_i(\mathbf{X}_i)$ has a lower bound $\lambda_{\min}$. For any $\delta > 0$, if we pick $\rho > (\delta - \lambda_{\min})/\min_{i,j}|B_{i,j}|$, then the subproblem defined in (8), (9) is strongly convex with modulus at least δ. The subproblem defined in (10) is strongly convex with modulus at least ρ (since the final term is a quadratic function with the second order coefficient being $\rho/2$), i.e., $\gamma_0(\rho) \geq \rho$. Thus the first sub-assumption holds. Since $\gamma_0(\rho) > \rho$, we only need to pick $\rho > \max\{\sqrt{2}L, 2L^2\}$ to satisfy the second sub-assumption $\rho > \max\{2L^2/\gamma_0(\rho), 2L^2\}$.

Assumption C3 requires that the objective function is lower bounded. In our problem, the objective function is non-negative, thus this assumption holds.

Assumption C4 requires that g_i's are smooth functions (possibly non-convex) and are Lipschitz smooth, i.e., there exists L_i such that

$$\|\nabla g_i(\mathbf{X}_i) - \nabla g_i(\tilde{\mathbf{X}}_i)\| \leq L_i\|\mathbf{X}_i - \tilde{\mathbf{X}}_i\|, \forall \mathbf{X}_i, \tilde{\mathbf{X}}_i \in \mathcal{X}_i. \tag{19}$$

As we are using smooth activation functions in the neural networks, the neural network mappings $\mathcal{N}_{met}$ and $\mathcal{N}_{mm}$ are both smooth functions of the input. The function g_i consists of two terms, each of which is a concatenation of the quadratic loss function and a neural network mapping, thus g_i is smooth. Since the feasible set $\mathcal{X}_0$ is a bounded set, the maximum eigenvalue of $\nabla^2 g_i$ has an upper bound L_i in the feasible set. Thus there exists L_i such that (19) holds.

Now we have verified Assumption C in [1]. Applying [1, Theorem 3.4], we obtain the desired convergence result.

Numerical Phantom Generation

Segmented brain compartments and tissue fraction maps, i.e., gray matter (GM), white matter (WM), and cerebrospinal fluid (CSF), were first obtained from an experimentally acquired brain T_1-weighted MPRAGE image (by SPM12, https://www.fil.ion.ucl.ac.uk/spm/). Compartmental concentrations and T_2^*/linewidths for the metabolites and MMs were assigned by adapting literature values [2–4]. More specifically, for metabolites (NAA, Glu, Cr1, Cr2, mI, Gln, Cho, GABA, GSH, and Lac), the concentrations (c_m) in the GM were 12.25, 9.25, 7.5, 4.25, 6.5, 9.25, 2.5, 1.5, 2.25, and 0.6 mM, respectively. And the T_2^*s were 250, 80, 150, 150, 150, 80, 200, 80, 70, and 220 ms, respectively. The concentrations for the WM were decreased by 30% with the T_2^* randomly decreased by 15%-20% for different molecules. In addition, the concentrations for all molecules in the CSF were assigned to be 0.1% of the concentrations in GM. For MMs (peaks located at 0.9, 1.21, 1.38, 1.63, 2.01, 2.09, 2.25, 2.61, 2.96, 3.11, 3.67, 3.8, and 3.96 ppm), the relative concentrations coefficients (b_l) in the GM were 0.72, 0.28, 0.38, 0.05, 0.45, 0.36, 0.36, 0.04, 0.2, 0.11, 0.64, 0.07, 1, respectively. And the linewidths (W_l) were 21.2, 19.16, 15.9, 7.5, 29.03, 20.53, 17.89, 5.3, 14.02, 17.89, 33.52, 11.85, 37.48 Hz, respectively. Similarly, the concentrations coefficients for the WM were decreased by 30% with the linewidths remains the same for different MMs. And the concentrations for all MMs in the CSF were also assigned to be 0.1% of the concentrations in GM. Next, a global scaling factor (similar to [4], on top of the individual coefficieint variations) for MM signals were introduced to reflect experimentally observed metabolite-to-MM signal ratios. The regional concentrations and linewidths were subsequently combined using the tissue fraction maps as weightings in a voxel-by-voxel fashion, generating continuously varying concentration and linewidth maps. A lesion-like feature was generated in the image with a significantly altered metabolite ratio, i.e., a factor of 3 higher Cho concentration and a factor of 3 lower concentrations for other metabolites compared to the GM. Finally, the simulated metabolite basis ($\{v_m\}$) and the spectral parameters at different voxels were fed into Eq. (2) to synthesize FIDs at individual voxels. To mimic practical scenarios, we also simulated voxel-dependent random frequency shifts for different molecules (with mean zero and a standard deviation of 5 Hz) as well as residual B_0 field inhomogeneity (mean zero and a standard deviation of 10 Hz). Noisy data were simulated by adding complex white Gaussian noise. The SNR is defined as the ratio between the maximum NAA peak amplitude (across the NAA map) and noise standard deviation (σ):

$$\mathrm{SNR} = \frac{\max_{\mathbf{r}} |\rho(\mathbf{r}, f_{NAA})|}{\sigma},$$

where f_{NAA} denotes the NAA peak frequency at around 2 ppm.

Supplementary Figures

This section shows the supplementary results mentioned in the main text.

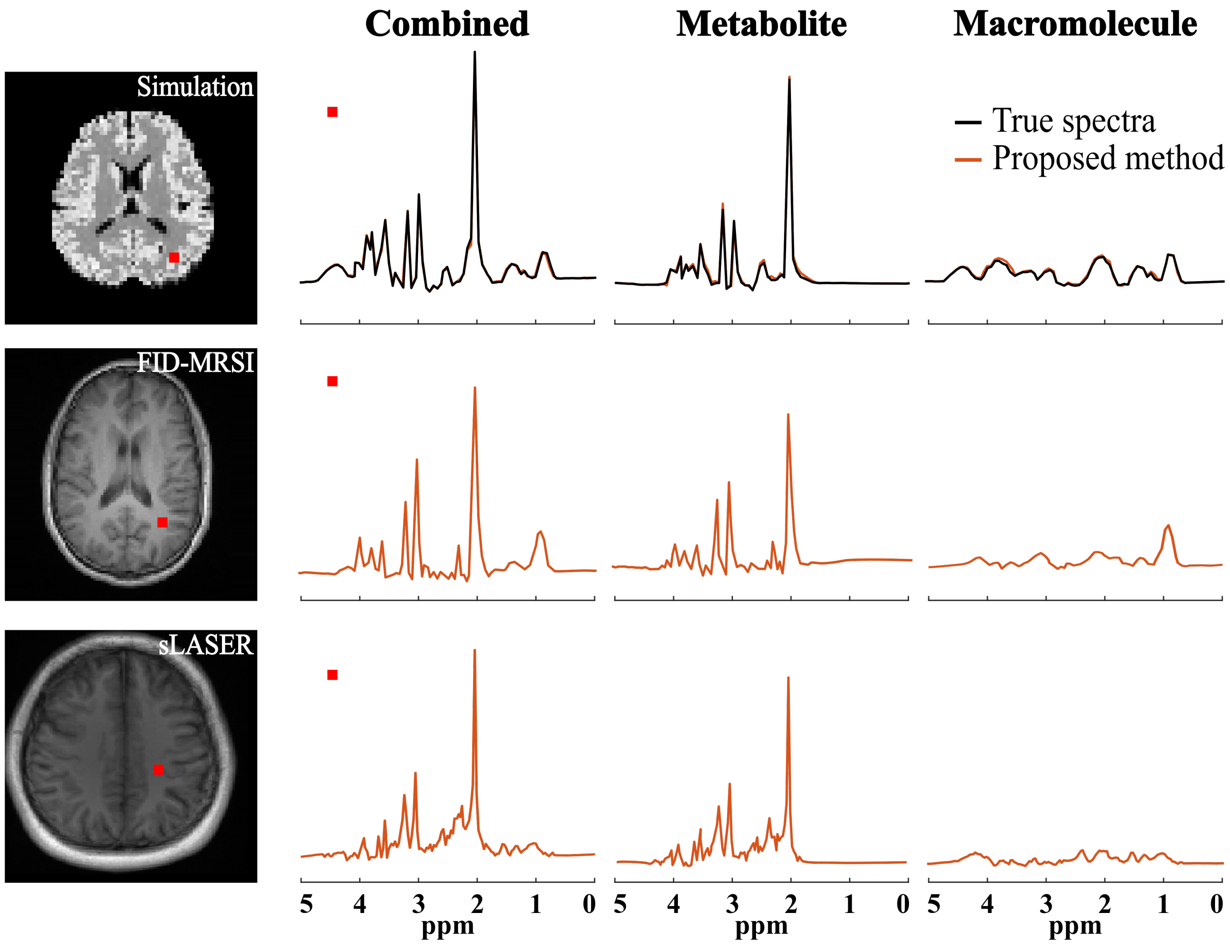

Figure S1: Reconstructed and separated spectra produced by the proposed method from the same data used in Figs. 3-7 with the real parts of the spectra shown (phase corrected). The voxel locations are marked (red squares) in the anatomical images on the left. The first row shows the results from the simulation phantom, while the second and third rows show spectra from the in vivo FID-MRSI and sLASER data, respectively.

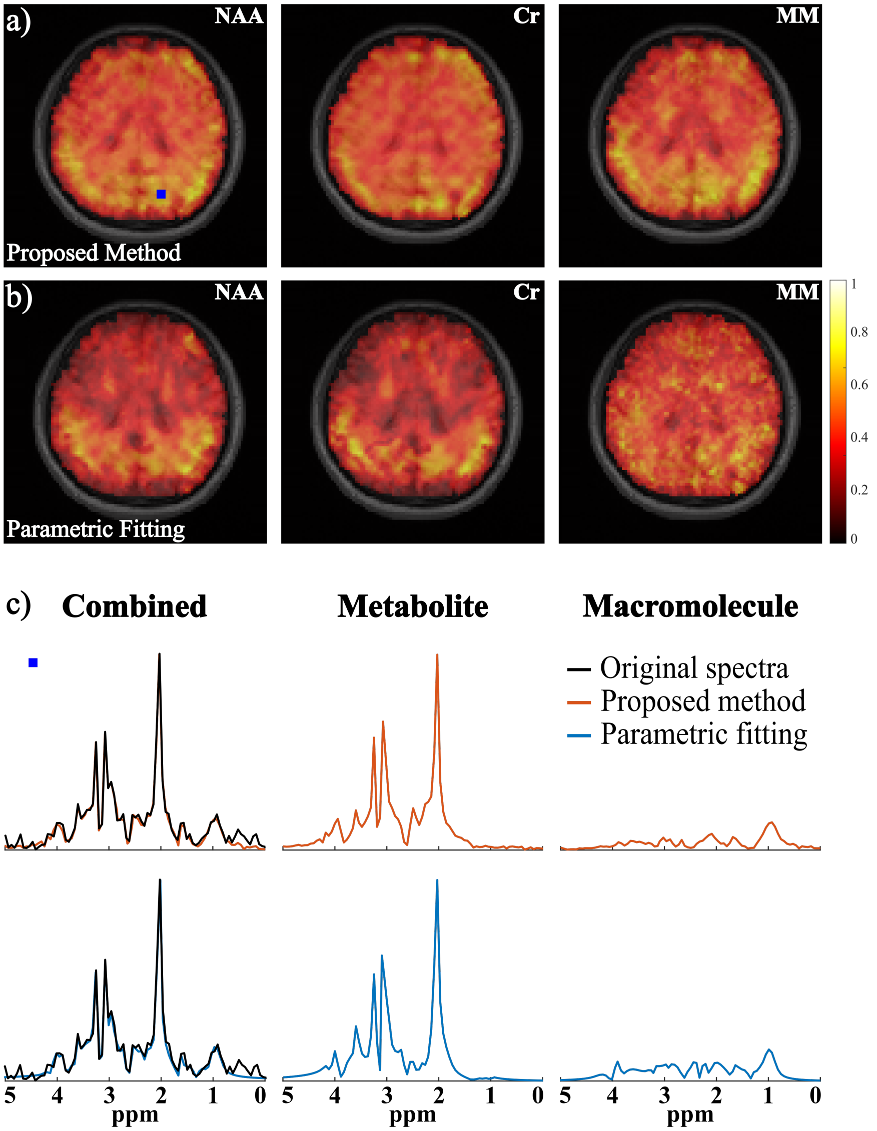

Figure S2-1: Experimental results from another volunteer (data acquired using the FID-MRSI sequence with TR/TE = 1500/4 ms, FOV = 220×220 mm^2, slice thickness = 10 mm, matrix size = 24×24, spectral bandwidth = 2000 Hz, and 512 FID samples): a) and b) Maps of NAA, Cr, and MM estimated from the separated signals produced by the proposed method (a) and the direct parametric fitting method (b). The maps were overlaid on an anatomical image for the matched slice; c) Spatially-resolved spectra (voxel location marked by the blue symbol) with the first and second rows showing the results from the proposed method (orange) and parametric fitting (blue), respectively. The original spectra are shown in black. A similar comparison to other data can be observed, with the results from the proposed method showing less spatial artifacts and less spectral under/over estimation.

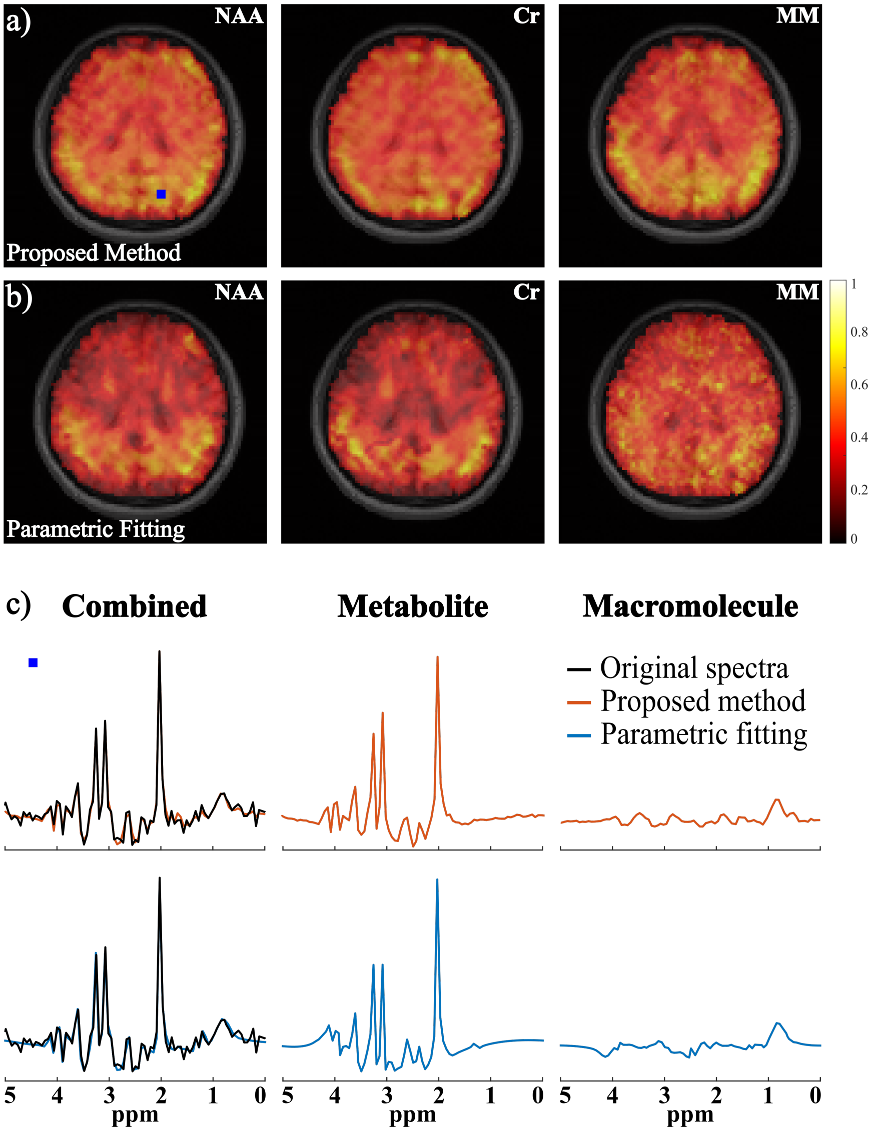

Figure S2-2: Same as Fig. S2-1 with spectra shown in real parts.

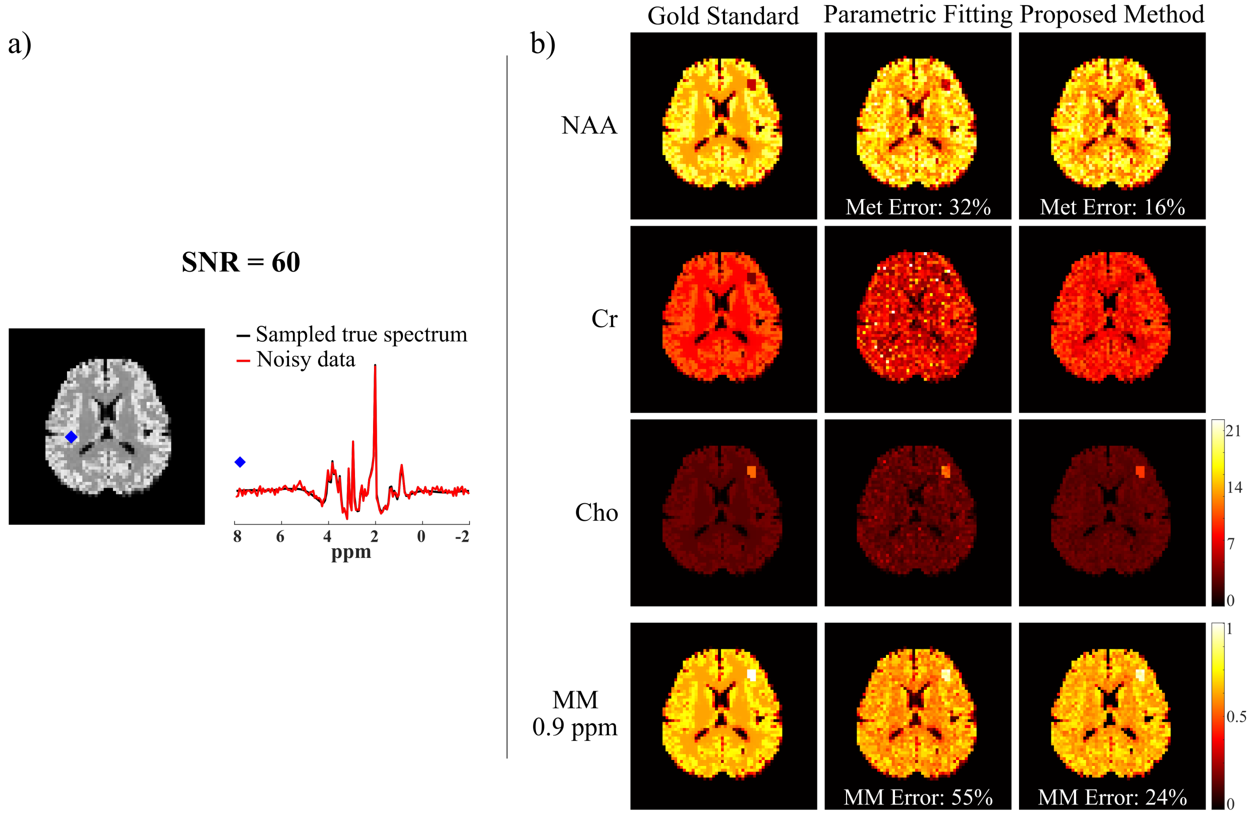

Figure S3: Simulation results from data with a higher SNR (SNR=60) a) A representative spectrum (real parts) from the phantom illustrating the noise level; b) Mapping results from the data: maps of NAA, Cr, Cho, and MM ($\sim$0.9 ppm peak) from the ground truth (Gold Standard, column 1), the direct parametric fitting method (column 2), and the proposed method (column 3) are compared, along with relative ℓ_2 errors for the separated metabolite and MM signals (shown in images). With the higher SNR, reduced spatial variances and lower errors can be observed, as expected.

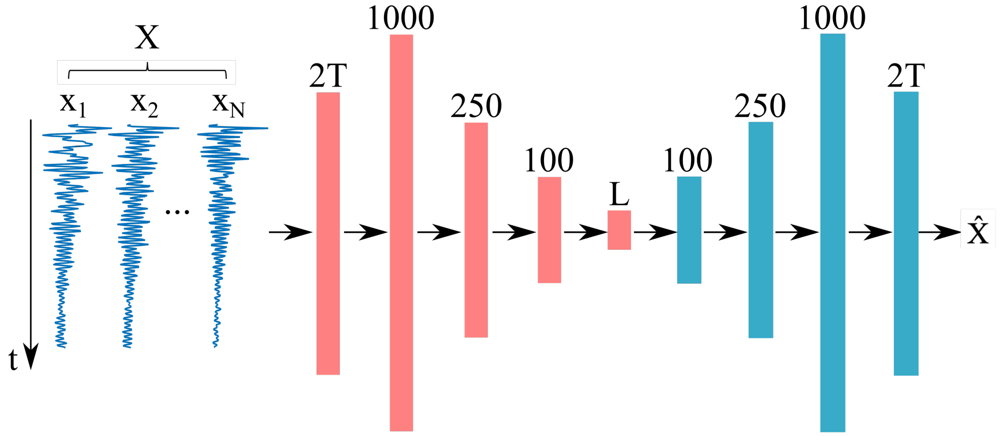

Figure S4: The DAE structure used. **X** denotes the collection of FID training data (each with a length T). The real and imaginary parts of these data are concatenated (thus a dimension of $2T$) to be used as input to the network. The DAE uses a fully connected "bottleneck" structure with an encoder (red) and a decoder (blue), and a middle feature layer with a dimension L (also referred to as the model order for our learned representations).

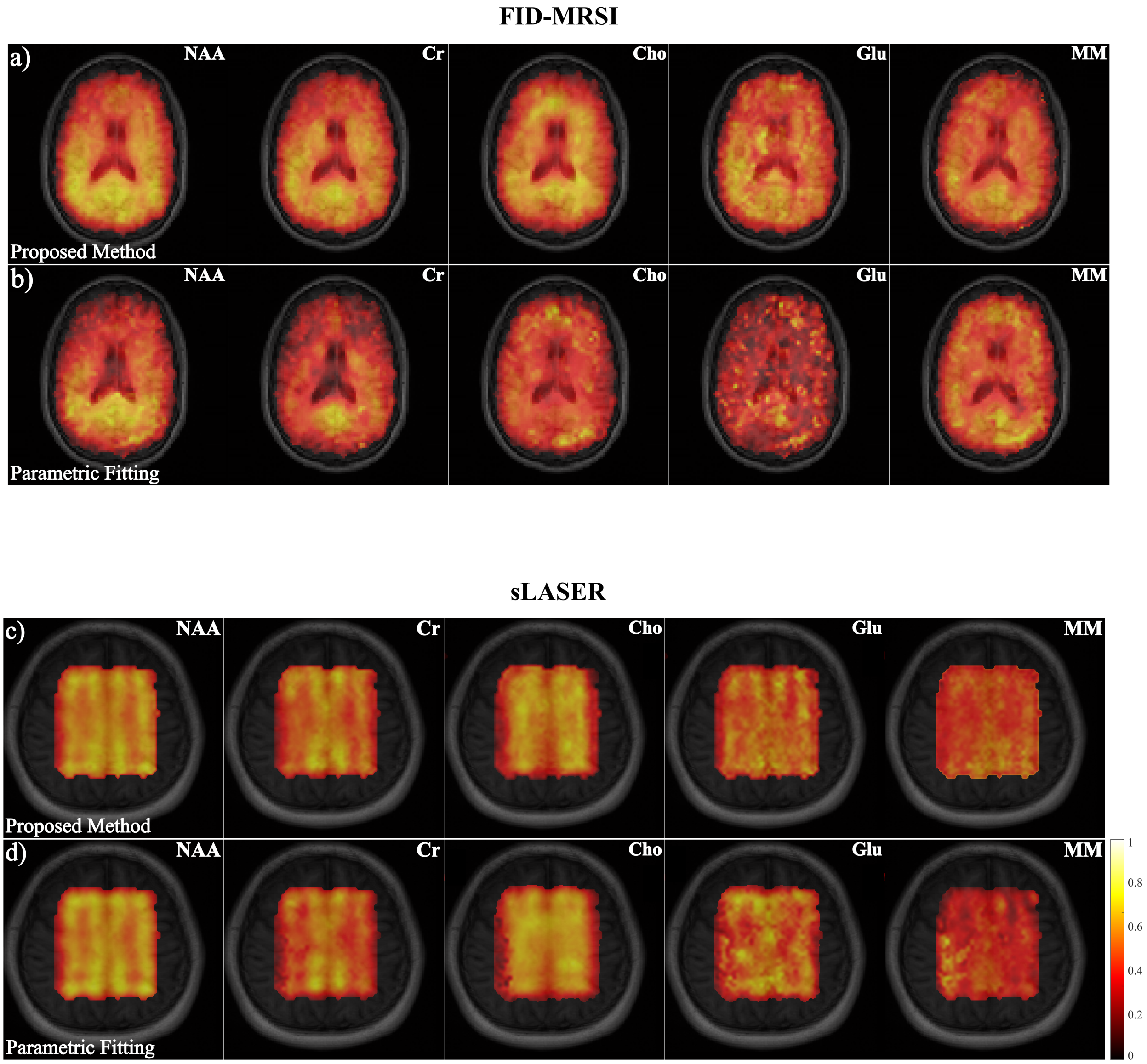

Figure S5: Experimental results from the in vivo FID-MRSI (top panel) and sLASER data (bottom panel). The top rows in each panel contain the molecular maps (NAA, Cr, Cho, Glu and MM) estimated from the separated signals produced by the proposed method (a and c), while the second rows in each panel the direct parametric fitting results (b and d). As can be seen, all the maps from the proposed method exhibit substantially less artifacts than those from the direct fitting, consistent with the comparison shown in the main text.

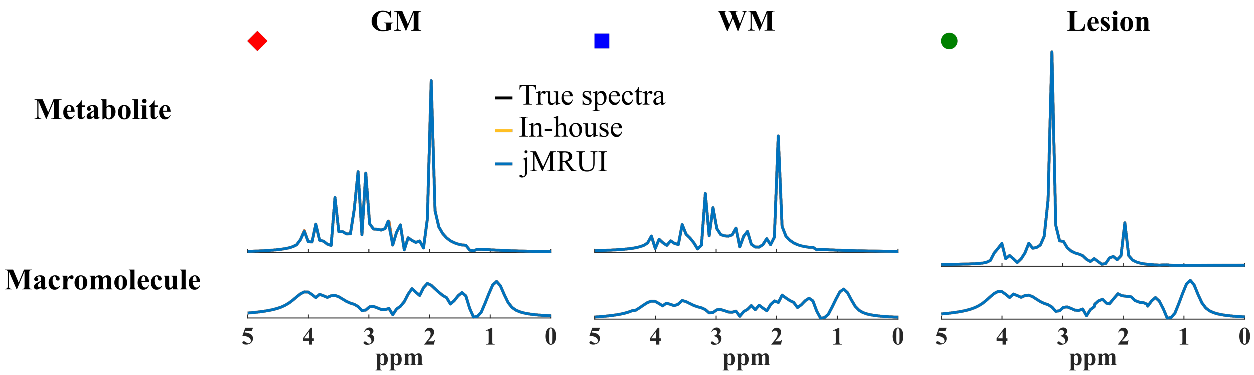

Figure S6: Comparison of the in-house fitting implementations against that provided by the jMRUI package. Different metabolite and MM spectra were simulated using experimental spectral parameters. As can be seen, our implementation produced almost the same results as those from jMRUI.

References

[1] M. Hong, Z.-Q. Luo, and M. Razaviyayn, "Convergence analysis of alternating direction method of multipliers for a family of nonconvex problems," *SIAM J. Optimz.*, vol. 26, pp. 337–364, 2016.

[2] R. A. de Graaf, *In vivo NMR spectroscopy: Principles and techniques.* John Wiley & Sons, 2019.

[3] R. Birch, A. C. Peet, H. Dehghani, and M. Wilson, "Influence of macromolecule baseline on 1H MR spectroscopic imaging reproducibility," *Magn. Reson. Med.*, vol. 77, pp. 34–43, 2017.

[4] H. H. Lee and H. Kim, "Intact metabolite spectrum mining by deep learning in proton magnetic resonance spectroscopy of the brain," *Magn. Reson. Med.*, vol. 82, pp. 33 – 48, 2019.
